# Evolutionary perspective of Big tau structure: 4a exon variants of MAPT

**DOI:** 10.3389/fnmol.2022.1019999

**Published:** 2022-12-02

**Authors:** Itzhak Fischer

**Affiliations:** Department of Neurobiology and Anatomy, Drexel University College of Medicine, Philadelphia, PA, United States

**Keywords:** tau protein, MAPT gene, microtubule-associated protein, exons, sequence homology, alternative splicing, Ensembl, nervous system

## Abstract

The MAPT gene encoding the microtubule-associated protein tau can generate multiple isoforms by alternative splicing giving rise to proteins which are differentially expressed in specific areas of the nervous system and at different developmental stages. Tau plays important roles in modulating microtubule dynamics, axonal transport, synaptic plasticity, and DNA repair, and has also been associated with neurodegenerative diseases (tauopathies) including Alzheimer’s disease and frontotemporal dementia. A unique high-molecular-weight isoform of tau, originally found to be expressed in the peripheral nervous system and projecting neurons, has been termed Big tau and has been shown to uniquely contain the large exon 4a that significantly increases the size and 3D structure of tau. With little progress since the original discovery of Big tau, more than 25 years ago, we have now completed a comprehensive comparative study to analyze the structure of the MAPT gene against available databases with respect to the composition of the tau exons as they evolved from early vertebrates to primates and human. We focused the analysis on the evolution of the 4a exon variants and their homology relative to humans. We discovered that the 4a exon defining Big tau appears to be present early in vertebrate evolution as a large insert that dramatically changed the size of the tau protein with low sequence conservation despite a stable size range of about 250aa, and in some species a larger 4a-L exon of 355aa. We suggest that 4a exon variants evolved independently in different species by an exonization process using new alternative splicing to address the growing complexities of the evolving nervous systems. Thus, the appearance of a significantly larger isoform of tau independently repeated itself multiple times during evolution, accentuating the need across vertebrate species for an elongated domain that likely endows Big tau with novel physiological functions as well as properties related to neurodegeneration.

## Introduction

**Figure 1:**
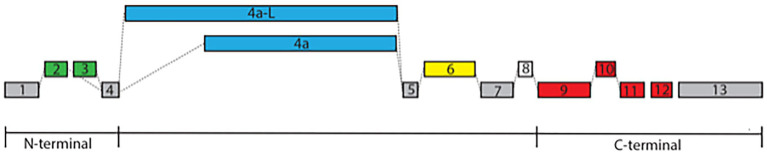
Exon structure of MAPT. The exon structure of MAPT derived from the Ensemble and NCBI databases includes alternative splicing at the N-terminal with either exons 2 and 3 or exon 2 alone labeled in green (exon 3 is always associated with exon 2), exon 4a or its longer form of 4a-L defining Big tau labeled in blue, exon 6 with 3 splice sites (6c, 6p, and 6d) labeled in yellow, exon 8 labeled in white, and exon 10, which is one of the MTBD defining the 3R/4R isoforms labeled in red. The constitutive exons 1, 4, 5, 7, and 13 are labeled in gray. Note that these alternative splicing can generate multiple isoforms with distinct temporal and spatial distributions.

The MAPT gene located on human chromosome *17q21* encodes the microtubule-associated protein tau, which is expressed primarily in the nervous system and neurons. MAPT produces multiple isoforms by alternative mRNA splicing of distinct exons (exons 1–13, Figure 1), giving rise to tau proteins which are differentially expressed in the nervous system, depending on the stage of neuronal maturation and the type of neurons (Kosik et al., 1989; Ruiz-Gabarre et al., 2022). In most regions of the human brain, tau is present as 6 distinct isoforms that vary in the expression of exon 2, 3 at the N-terminal, and at the C-terminal exon 10, one of the microtubule-binding domains (MTBD), defining isoforms with 3 and 4 repeats (3R and 4R respectively; Andreadis, 2005; Trabzuni et al., 2012).

Tau has been traditionally studied in the context of mediating the properties of microtubules (MT) through its MTBD at the C-terminal, which is shared with the other members of the MAP 4/MAP2/MAPT family (Dehmelt and Halpain, 2005; Baas and Qiang, 2019). It has also been shown to play roles in the interaction of MTs with other cytoskeletal proteins and the membrane, as well as axonal transport and synaptic plasticity. These properties underscore the importance of the N-terminal domain, which projects from the MT surface to allow interaction with other cellular structures (Himmler et al., 1989; Li et al., 2003; Brandt et al., 2020). Tau’s clinical significance has been shown in a variety of neurodegenerative diseases (tauopathies) including Alzheimer’s disease, Pick’s disease, frontotemporal dementia, cortico-basal degeneration, and progressive supranuclear palsy (Chang et al., 2021; Song et al., 2021). The complex degeneration process of neurons associated with tau is mediated by mutations in the MAPT gene as well as aggregation and hyperphosphorylation of the tau protein (Lee and Leugers, 2012; Wang and Mandelkow, 2016).

What has been less studied is the high-molecular-weight (HMW) isoform, termed Big tau, which contains a large exon termed 4a that increases the MW of tau from the range of 45–65 kDa to 110 kDa (Georgieff et al., 1991). This Big tau isoform is expressed mainly in the peripheral nervous system (Couchie et al., 1992), but also in distinct regions of the central nervous system that project to the periphery (Boyne et al., 1995; Nunez and Fischer, 1997). The expression of tau in dorsal root ganglion (DRG) neurons switches from the LMW isoforms at early stages to Big tau in the adult represented by a mRNA of 8 kb size (Oblinger et al., 1991; Boyne et al., 1995). We summarized these data in a recent review (Fischer and Baas, 2020) and expressed our surprise that there has been little progress since the original discovery, more than 25 years ago, about the structure and function of Big tau that could shine light on the switch to Big tau and its evolutionary origin.

In this study, we searched the structure of the MAPT gene against available databases to analyze the exon composition of Big tau variants as they evolved from early vertebrates to primates and human (Gottschalk and Hefti, 2022; Ruiz-Gabarre et al., 2022). Our objective was to compare the conservations of the protein sequences with focus on variants of the 4a exon, and to generate a comprehensive analysis of the homology during the evolution of Big tau relative to humans. We discovered that the 4a exon defining Big tau appears to be present early in vertebrate evolution as a large insert that dramatically changed the size of the tau protein and likely its 3D structure. There was, however, low sequence conservation of the 4a exon despite a stable size range of about 250aa, and in some species a similar but larger 4a-L exon of 355aa (Souter and Lee, 2010). We discuss the possibility that 4a exon variants evolved independently in different species by exonization using new alternative splicing. We also argue that the need for differential expression of specific isoforms of tau and their localization in defined regions may have increased with the growing complexities of the evolving nervous systems (Andreadis, 2005; Trushina et al., 2019). Big tau may therefore be an example of a rather dramatic modification of tau structure that evolved multiple separate times during evolution to satisfy biological needs that the other variants could not support effectively.

## Materials and methods

### Databases

For analysis of exon structure of the microtubule-associated protein tau (MAPT) gene in the vertebra subphylum and its 5 major classes, we used Ensembl as a genome browser (Ensembl Release 106, Apr 2022). The Ensembl Genome project is based at the European Molecular Biology Laboratory’s European Bioinformatics Institutes (EMBL-EBI), with the mission of generating gene/protein trees with predicted structure including putative exons. We primarily used the MAPT GeneTree ENSGT00940000155494 that contains 262 genes and 224 speciation nodes with protein sequences annotated for predicted exons (shown in alternate black and blue letters). The gene trees are generated by the Gene Orthology/Paralogy prediction method pipeline. Gene trees are constructed using one representative protein for every gene in every species in Ensembl. The longest translation annotated by the CCDS project is used, if any are available, or the longest protein-coding translation otherwise. These trees are reconciled with a species tree, generated by TreeBeST. In parallel, we also used the Ensembl list of 255 orthologs to the human MAPT which contained the full transcript table for each species (e.g., 8 transcripts for the chimpanzee MAPT ENSPTRG00000009314) as well as alignment of its canonical transcript against human MAPT.^1^

To gain the broadest record of tau sequences containing the 4a exon variants we also searched the NCBI Ortholog for MAPT, which contained 257 genes for tetrapod (*Tetrapoda*) at https://www.ncbi.nlm.nih.gov/gene/4137/ortholog/?scope=32523 including birds, turtles, alligators, lizards, mammals, and amphibians. Ortholog gene groups are calculated by NCBI’s Eukaryotic Genome Annotation pipeline for the NCBI Gene dataset using a combination of protein sequence similarity and local synteny information. Only genes in the NCBI Gene database are in scope for ortholog calculation which is currently limited to vertebrates. The list of the ortholog MAPT genes is linked to gene and protein sequences.

At later stages of the project, we searched the recently “Highly accurate protein structure prediction with AlphaFold” (Jumper et al., 2021). The AlphaFold database was created by DeepMind and EMBL’s European Bioinformatics Institute (EMBL-EBI) to make these predictions freely available to the scientific community. The latest database release contains over 200 million entries that allowed easy reference to UniProt and helped us verify the database we used for the analysis.

For comparison with other microtubule-associated proteins (MAP4, MAP2, and MAPT), we used the OrthoDB v10.1 from the Swiss Institute of Bioinformatics for the hierarchical catalog of orthologs.^2^

### Alignments and homology

For pairwise sequence alignment, we used the EMBL-EBI EMBOSS Needle program, which is using the Needleman–Wunsch global alignment. For multiple sequence alignment (MSA), we used the Clustal Omega as a tool that is using seeded guide trees and HMM profile-profile techniques to generate alignments. This program generated not only alignments to assign identity and similarity relative to the human MAPT but also a percent identity matrix for a comprehensive comparison among members of the search. We also used the NCBI program of COBALT as a MSA tool which accepts protein accessions or FASTA formats and simultaneously aligns multiple protein sequences utilizing information about protein domains with a balanced compromise between speed and accuracy. COBALT allowed us to view not only the sequence alignment but also a graphical overview and a phylogenic tree. For further analysis of possible homology of the 4a exon or the extra sequences of 4a-L we used BLASTP searches followed by alignment and taxonomy analysis. Although we did not perform extensive statistical analysis, in general terms, for this study, we interpreted as significance identities that were >20%. At a lower homology score, when the alignment was scattered without distinct areas of contiguous identity, we classified these cases as lacking important homology. As an additional resource for comparison with the human MAPT gene, we occasionally used the HUGO Gene Nomenclature Committee (HGNC) reports at https://www.genenames.org/data/gene-symbol-report/#!/hgnc_id/HGNC:6893.

## Results

Searching the MAPT gene with Ensembl as a genome browser as well as the NCBI gene bank generated multiple protein sequences for each of the species we analyzed. We aimed to use the Ensemble “canonical transcript” defined as the most conserved, mostly highly expressed, has the longest coding sequence, and is represented in other key resources such as NCBI and UniProt. However, we often searched and included additional sequences to identify the various 4a exon variants. The complete list of MAPT sequences used for the analysis with full annotations and manual exon assignments is shown in Supplementary Figure 1. For some species, we included multiple MAPT sequences (Human, Chimpanzee, Gibbon, Gorilla, Baboon, Lemur, Rat, Dog, and Carp). In a few cases (rat, opossum) we corrected the exon annotation predicted by Ensembl, and in species of early evolutionary stages (e.g., jawless fishes) the exon assignment at the N-terminal exons remained uncertain. As our aim was to compare the conservations of the exon sequences with focus on variants of the 4a exon, rather than investigate the expression pattern, we often chose representative MAPT genes that includes the 4a exon and whenever possible the MAPT gene with the longer 4a-L form. Indeed, a BLAST search of the unique human 4a-L, which was comprised of an addition of 104aa over 4a, revealed a list which included mostly primates and some carnivores. However, using the BLAST search was of limited use beyond primates and mammal because of low conservation. To identify the 4a and 4a-L exons in vertebrates (birds, reptiles, amphibian, and fish) we had to use manual curation for a long exon located between exons 4 and 5.

All the homology analysis from primates to fishes was calculated in comparison to the human MAPT, but we also provided data in identity matrix tables that showed systematic homology comparison among the different species (e.g., Tables 1B,C). Human MAPT has multiple genomic forms in Ensembl and multiple transcripts in NCBI. We therefore used 2 representative forms: the 776aa homo sapiens MAPT (ENST00000415613.6 MAPT-205), which contains the 4a composed of 251aa, and the canonical 833aa transcript which contains the 4a-L of 355aa (ENST00000262410.10 MAPT-201), as shown in Supplementary Figure 1.

**Table 1:** Analysis of MAPT in primates.

		**Human**		**Chimp**		**Gorilla**	**Gibbon**	**Baboon**		**Marmoset**	**Lemur**
**Panel A**											
**MAPT**	Identities (%)	100	100	99	99	99	97	94	92	85	81
	Similarities (%)			99	96	99	98	94	93	88	84
	Size (aa)	776	833	776	833	776	756	780	851	852	767
**Exons 1–4 (N Terminal)**		100	100	100	100	98	97	95	95	90	85
**Exon 4a**	Identities (%)	**100**	**99**		**98**	**94**	**90**				**64**
	Size (aa)	**251**		**251**		**251**	**251**	**251**			**251**
**Exon 4a-L**	Identities (%)		**100**		**98**	**98**			**90**	**81**	
	Size (aa)		**355**		**355**	**353**			**355**	**355**	
**Exons 9–13 (C-Terminal)**		100	100	100	100	100	100	99		99	98
**Panel B**											
**Exon 4a**											
Human 776		100	99	98	94	90	69				
Chimp		99	100	98	94	90	69				
Gorilla		98	98	100	94	90	69				
Gibbon		94	94	93	100	88	68				
Baboon		90	90	90	88	100	69				
Lemur		68	69	68	69	68	100				
**Panel C**											
**Exon 4a-L**											
Human 833		100	99	98	90	81					
Chimp		99	100	97	89	80					
Gorilla		98	97	100	89	80					
Baboon		90	89	89	100	80					
Marmoset		81	80	80	80	100					

Panel A: MAPT sequence homology was analyzed against human (776aa and 833aa) for chimpanzees (776aa and 833aa), gorilla (776aa), Gibbon (756aa), baboon (780aa and 851aa), marmoset (852aa), and lemur (767aa) all taken from the database presented in Supplementary Figure 1. It included homology (identity and similarity) for the complete MAPT sequence relative to human MAPT, exons 1–4 representing the N-terminal, exon 4a, exon 4a-L and exons 9–13 representing the C-terminal including the MTBD. Panel B: matrix identity for exon 4a. Panel C: matrix identity for exon 4a-L.

### Primates

**Figure 2:**
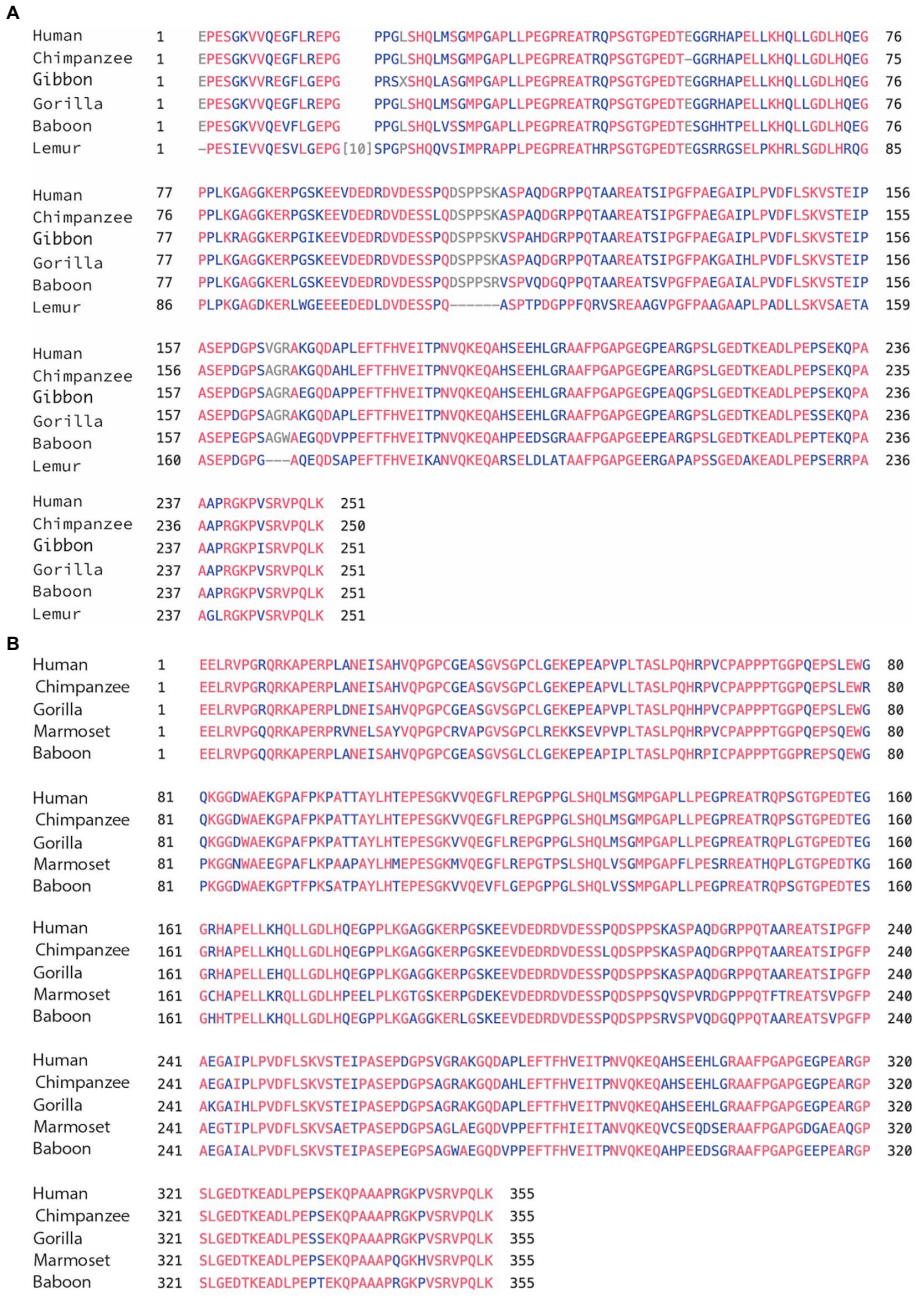
**(A)** Alignment of exon 4a in primates. Using the multiple alignment tool of COBALT for analysis of the 4a exon in primates including human, chimpanzee, gibbon, gorilla, baboon, and lemur. **(B)** Alignment of exon 4a-L in primates. Using the multiple alignment tool of COBALT for analysis of the 4a-L exon in primates including human, chimpanzee, gorilla, marmoset, and baboon.

We used the 2 isoforms of human MAPT (the 776aa and the 833aa with 4a and 4a-L, respectively) as reference in comparison to MAPT of apes: chimpanzee, gorilla, Gibbon, monkeys (anthropoids): baboon, marmoset, and prosimians: lemur (Table 1A). We found very high sequence identity to human MAPT at 99%–97%: apes, 94%–85%: monkeys, and 81% lemur (sequence similarity was a little higher). The relatively low identity of 85% in marmoset is likely related to the branch of New World monkeys and similarly, the 81% identity in lemur is not surprising as it belongs to the family of strepsirrhine primates, 50 M years away from primates. Analysis of exons 1–4 of the N-terminal region (Supplementary Table 1a) has also shown high identity of 100%–85%, with exons 9–13 of the MTBD (Supplementary Table 1b) showing the highest identity of 100%–98%. Importantly, the 4a exon had a lower identity of 99%–94% with apes, up to 90% for monkey, and 64% with lemur (Table 1A). There was a similar level of relatively low homology when 4a-L was included in the analysis with 98% sequence identity for apes, and 90%–81% for monkeys (Table 1B). As shown in Table 1A, in every case the conservation of exons 4a/4a-L was lower than any of the other measures (total MAPT, exons 1–4 or exons 9–13). Using a Blast search with the extra 104 aa of exon 4a-L, we found that surprisingly it was present in most primates. We also explored the presence of exon 8, which is rarely if ever expressed, and found it in most of the predicted genomic sequences of primates, but not all. For example, the 776aa variant of human MAPT containing 4a includes exon 8, while the 833aa variant containing 4a-L lacks exon 8. The overall graphical alignment of exon 4a is shown in Figure 2A using the Cobalt NCBI program and for exon 4a-L in Figure 2B.

### Mammals

**Figure 3:**
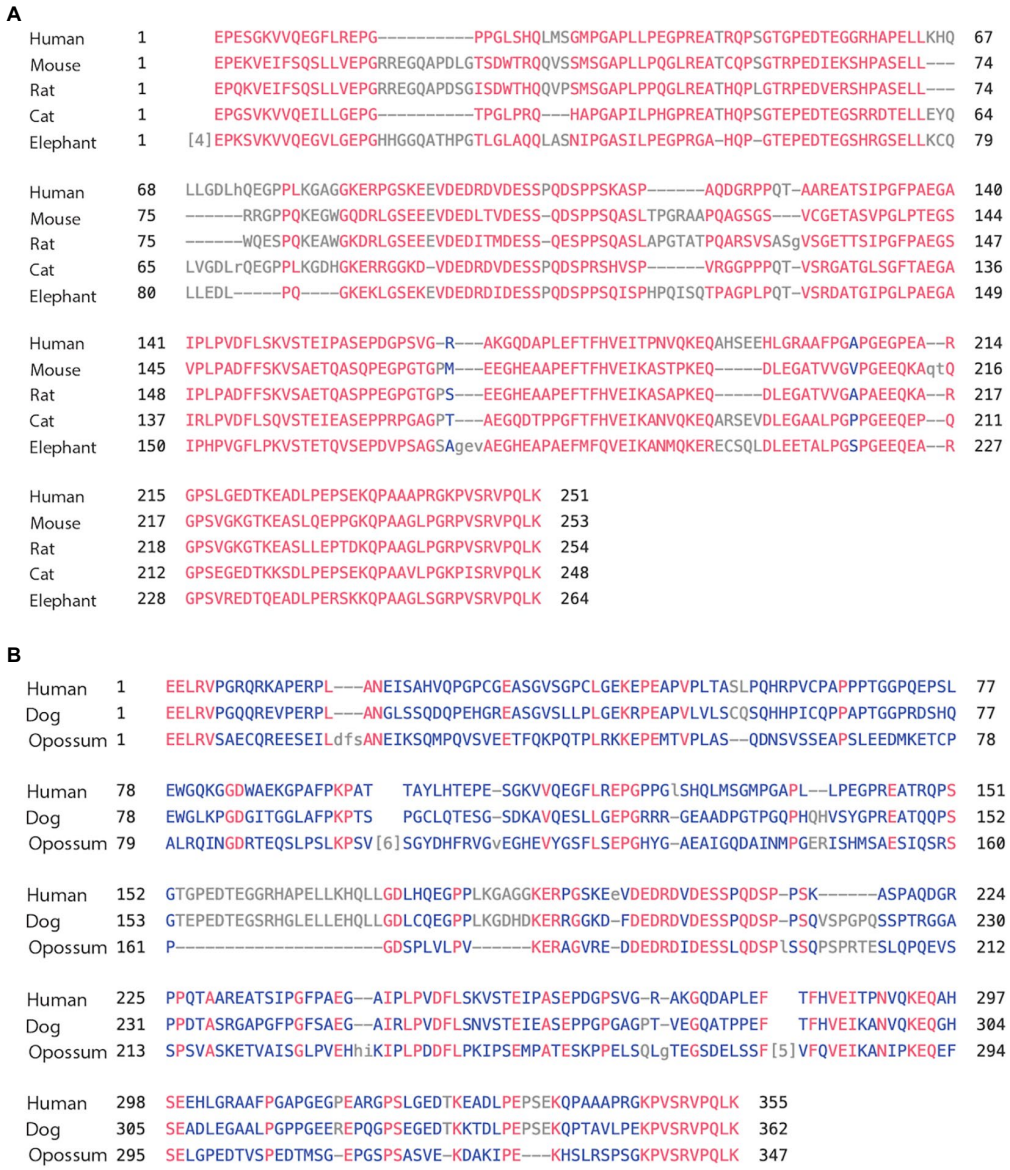
**(A)** Alignment of exon 4a in mammals. Using the multiple alignment tool of COBALT for analysis of the 4a exon in mammals including human, mouse, rat, cat, and elephant. **(B)** Alignment of exon 4a-L in mammals. Using the multiple alignment tool of COBALT for analysis of the 4a-L exon in mammals including human, dog, and opossum.

We again compared the human MAPT gene structures to rodents (Glires): rat and mouse, carnivores (Laurasiatheria): dog and cat, herbivore (afrotheria): elephant, and marsupial: opossum. As shown in Table 2A, the overall homology of MAPT was lower than primates and stood at a range of 84%–71% sequence identity with the marsupial opossum at 46%. Exons 1–4 of the N-terminal had 87%–83% identity, with opossum at 64%, consistently higher than total MAPT (Supplementary Table 2a). As expected, exons 9–13 of the MTBD had a very high sequence identity of 99%–97% and even the opossum showed a 94% identity (Supplementary Table 2b). In contrast, exons 4a/4a-L had a much lower sequence identity of 70%–64% (Dog, cat, elephant), 56% rodents, and 39% opossum (Tables 2B,C). The average sequence identity of the 4a/4a-L exons in mammal was 58% lower than the average of 87% in primates. There were still examples of the 4a-L exon, represented in our analysis with the dog and opossum. We had also shown the presence of exon 8 using Blast search in carnivores (e.g., cat). The overall graphical alignment of exon 4a in mammals is shown in Figure 3A using the Cobalt NCBI program for exon 4a-L in Figure 3B.

**Table 2:** Analysis of MAPT in mammal.

		**Human**		**Cat**	**Elephant**	**Dog**	**Rat**	**Mouse**	**Opossum**
**Panel A**									
**MAPT**	Identities (%)	100	100	84	75	75	71	72	46
	Similarities (%)			88	80	79	76	78	52
	Size (aa)	776	833	778	760	869	750	749	860
**Exons 1–4 (N Terminal)**			100	86	83	87	83	83	65
**Exon 4a**	Identities (%)	**100**		**70**	**65**		**56**	**56**	
	Size (aa)	**251**		**248**	**264**		**254**	**253**	
**Exon 4a-L**	Identities (%)		**100**			**64**			**39**
	Size (aa)		**355**			**362**			**347**
**Exons 9–13 (C-Terminal)**			100	98	99	99	97	97	94
**Panel B**									
Human 776		100	70	65	56	56			
Cat		70	100	65	53	55			
Elephant		65	65	100	54	54			
Rat		56	53	54	100	84			
Mouse		56	55	54	84	100			
**Panel C**									
**Exon 4a-L**									
Human 833		100	64	39					
Dog		64	100	35					
Opossum		39	35	100					

Panel A: MAPT sequence homology was analyzed against human (776aa and 833aa) for cat (778aa), elephant (760aa), dog (869aa), rat (750aa), mouse (749aa), and opossum (860aa) all taken from the database presented in Supplementary Figure 1. It included homology (identity and similarity) for the complete MAPT sequence relative to human MAPT, exons 1–4 representing the N-terminal, exon 4a, exon4a-L and exons 9–13 representing the C-terminal including the MTBD. Panel B: matrix identity for exon 4a. Panel C: matrix identity for exon 4a-L.

### Vertebrates (other than mammals)

**Figure 4:**
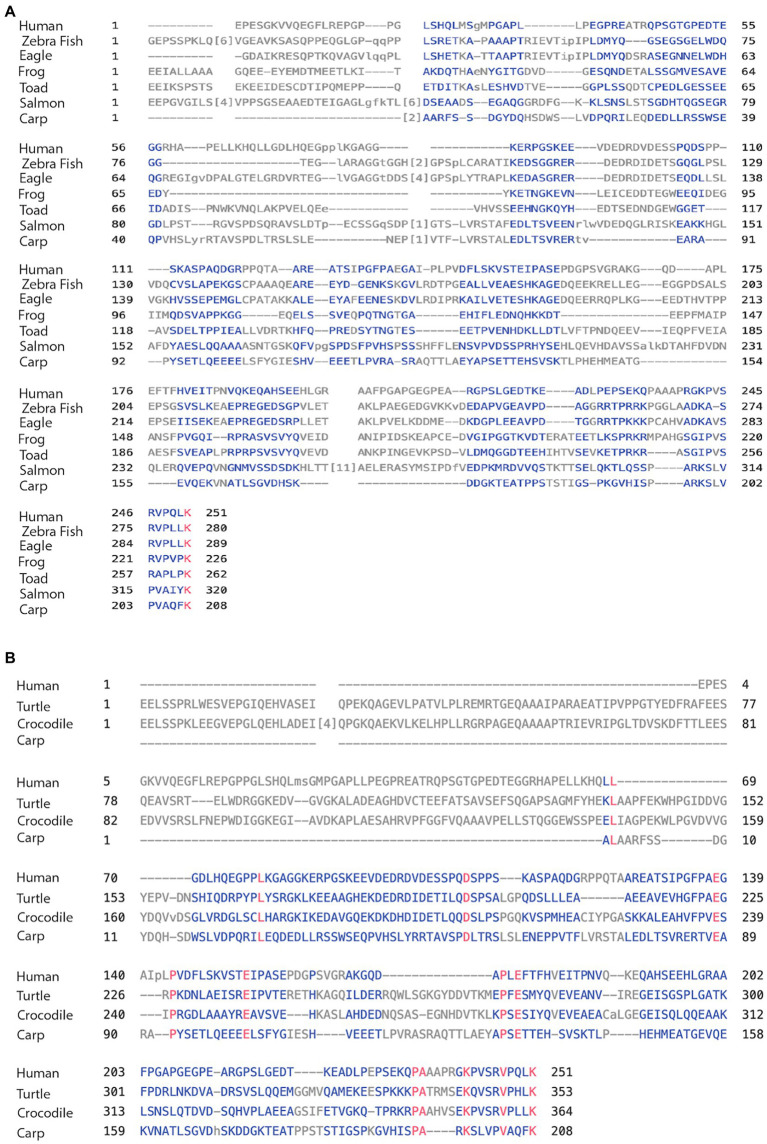
**(A)** Alignment of exon 4a in non-mammal vertebrates. Using the multiple alignment tool of COBALT for analysis of the 4a exon in vertebrates including human, zebra finch, eagle, frog, toad, salmon, and carp. **(B)** Alignment of exon 4a-L in non-primate vertebrates. Using the multiple alignment tool of COBALT for analysis of the 4a-L exon in vertebrates including human, turtle, crocodile, and carp.

Here, we compared the human MAPT gene structures to birds: zebra finch and golden eagle, reptiles: painted turtle and Australian saltwater crocodile, amphibians: the tropical clawed Xenopus frog, the Leishan spiny toad, and fish: Atlantic salmon and Common carp (Table 3A). The overall MAPT homology against human these vertebrates decreased to 52% sequence identity with birds, 52%–50% with reptiles, 43%–41% amphibians, and 30%–27% in fish. In fact, most of the overall homology was the result of high values at the MTBD of exons 9–12 and to a lesser extent the N-terminal of exons 1–4. Indeed, the MTBD of exons 9–13 had sequence identities of 93%–81% in birds, reptiles, and amphibians, and 55%–53% in fish (Supplementary Table 3b), while exons 1–4 of the N-terminal; maintained sequence identity of 62%–60% in birds and reptiles, decreased to 38%–32% in amphibians and was no longer significant in fishes at 20%–16% (Supplementary Table 3a). These results underscore the strong conservation of the MTBD relative to the slow evolution of the N-terminal discussed later. The homology of the 4a/4a-L exons was very low in all cases ranging from 28% to 15% sequence identity (Tables 3B,C) with just a few regions of homology as shown in the alignment of Figures 4A,B. To identify the putative 4a exons, which lacked homology, we needed to identify the adjacent exons. Indeed, we calculated the average 4a conservation in these vertebrate relative to the complete MAPT to be 49%. When compared to the MTBD the relative conservation value was only 27% representing the two extremes of lowest and highest regions of homology.

**Table 3:** Analysis of MAPT in vertebrates (non-mammalians).

			**Human**		**Zebrafish**	**Eagle**	**Turtle**	**Crocodile**	**Frog**	**Toad**	**Salmon**	**Carp**	
**Panel A**												
**MAPT**	Identities (%)	100	52	52	52	50	43	41	30	27	34	
	Similarities (%)			60	60	60	58	55	53	39	37	43
	Size (aa)	776	833	705	836	828	889	745	760	731	687	814
**Exons 1–4 (N Terminal)**			100	60	62	62	62	38	32	20	16	16
**Exon 4a**	Identities (%)	**100**		**24**	**26**			**16**	**15**	**17**	**15**	
	Size (aa)	**251**		**280**	**305**			**226**	**262**	**320**	**208**	
**Exon 4a-L**	Identities (%)		**100**			**28**	**25**					**18**
	Size (aa)		**355**			**353**	**364**					**400**
**Exons 9–13 (C-Terminal)**			100	91	93	92	92	80	81	55	53	53
**Panel B**												
**Exon 4a**												
Human 776		100	24	26	16	15	17	15				
Zebrafish		24	100	65	15	16	17	16				
Eagle		26	65	100	18	19	16	20				
Frog		16	16	18	100	31	17	19				
Toad		15	16	19	31	100	20	16				
Salmon		17	17	16	17	21	100	29				
Carp		15	16	20	19	16	29	100				
**Panel C**												
**Exon 4a-L**												
Human 833		100	28	25	18							
Turtle		28	100	45	17							
Crocodile		25	45	100	16							
Carp		18	17	16	100							

Panel A: MAPT sequence homology was analyzed against human (776aa and 833aa) for cat (778aa), zebra finch (705aa), eagle (836aa), turtle (828aa), crocodile (889aa), frog (745aa), toad (760aa), salmon (731aa), and carp (687aa and 814aa3) all taken from the database presented in Supplementary Figure 1. It included homology (identity and similarity) for the complete MAPT sequence relative to human MAPT, exons 1–4 representing the N-terminal, exon 4a, exon4a-L and exons 9–13 representing the C-terminal including the MTBD. Panel B: matrix identity for exon 4a. Panel C: matrix identity for exon 4a-L.

A separate analysis of jawless fishes including hagfish and lamprey was carried out only for the overall homology of MAPT because it was not possible identify the exon structure of 4a. The very low homology to human MAPT of 21% and 18% sequence identity, respectively, was under the threshold of meaningful homology, lower than that of bony fishes of about 30%. Interestingly, and as was previously reported, when compared to exons 9–13 containing the MTBD, the homology increased to 39% and 45% identity and 52/57% similarity for hagfish and lamprey, respectively (Table 4).

**Table 4:** Analysis of MAPT in jawless fish.

Tau	Human	Hagfish	Lamprey
Identities total (%)	100	21	18
Size (aa)	776	331	277
Identity with MTBD (%)	100	39	45

Panel A: MAPT sequence homology was analyzed against human (776aa) for hagfish (331aa), and lamprey (2777aa) all taken from the database presented in Supplementary Figure 1. It includes homology (identity and similarity) for the complete MAPT sequence relative to human MAPT, as well as the identity against the human MTBD of exons 9–13.

### Invertebrates

We included in our database (Supplementary Figure 1) a fly analog of tau from the Drosophila database composed of 361aa (isoform A) which had sequence identity/similarity with the human MTBD of 24/34% underscoring the evolutionally origin of the MAP4/MAP2/tau family as a microtubule-binding protein. Although we did not analyze the exon structure of this protein, it was reported to contain five putative MTBD repeats and a proline-rich N-terminal (Heidary and Fortini, 2001).

## Discussion

### Introduction

In our recent review about Big tau entitled “Resurrecting the mysteries of Big tau” (Fischer and Baas, 2020), we suggested several hypotheses for the dramatic structural difference between “conventional” LMW tau and Big tau. For example, we speculated that the inclusion of the large 4a exon in Big tau may provide protection from pathologically misfolding “as the much-increased length of the projection domain of Big tau may nevertheless affect the conformational changes or the formation of the abnormal filament.” Considering that tau is a naturally disordered protein which can adopt dynamic conformations (Fichou et al., 2015), it is possible that the 4a insert induces a transition to a more ordered state with reduced folding and aggregation properties. This transformation had both structural and functional consequences discussed below. Another possibility is that switching to Big tau in neurons with long axons typical of the PNS is associated with more efficient axonal transport either through interactions with motor proteins or providing larger spacers between neighboring MTs or affecting their assembly and bundling (Safinya et al., 2019). Indeed, an important feature of the 4a insert is the lack of extensive phosphorylation sites which are typical features of the rest of the tau protein suggesting a “functionally inert zone that provides length to the projection domain of tau.” To gain a better understanding for the “Big tau mysteries,” we focused this study on the evolution of the exon structure of MAPT and, with particular attention to the properties of the 4a exon variants.

### Methodology

What became evident as we started the analysis was that there were multiple protein sequences for each of the species. As noted in Methods, we used the canonical transcripts included in the MAPT GeneTree, which Ensembl defines as a representative transcript “most conserved, most highly expressed, has the longest coding sequence and is represented in other key resources such as NCBI and UniProt.” For example, for the baboon, there was a list of 19 isoforms.^3^ Herewe, used for the analysis of the 4a exon the MAPT transcript ENSPANT00000017634.3 generated by the Ensembl genome browser which had 780aa, and a 251aa of exon 4a, which is similar to human. For the analysis of 4a-L exon, we used isoform X1 (XP_017805564.2), which had 852aa with 355aa for 4a-L, again similar to human. In other species, where the different isoforms contained only the 4a exon, we started with the MAPT generated by Ensembl, but made sure that the sequence was a valid representative for the species we investigated. Similarly, when we compared the alignment results using the EMBL-EBI EMBOSS Needle or the Clustal Omega programs relative to the NCBI Cobalt program we found minor differences, which for the overall objective of this study did not change the general picture of the results or the conclusions. As mentioned in Methods, scattered identity of less than 20% was interpreted as nonhomologous. For example, the comparison of the 4a exons from reptilians (turtle, crocodile) and amphibian (frog, toad) to human showed 14%–17% identity all scattered throughout >200aa.

### Major findings

One of our main findings of the homology analysis in vertebrates across the different classes was the low sequence conservation of the 4a exon despite a stable size range of about 250aa, which represents not only the largest exon but a 50% increase relative to LMW tau isoforms. A similar stable size of about 350aa and low homology was found in the 4a-L exon variant.

While the conservation of the 4a exon was consistently low at 25%–17% sequence identity in non-mammal including birds, reptiles, amphibian, and fish, the homology increased in mammals (with few exceptions) for sequence identity of 50%–60% culminating in primates with identity of 90%–100%. As our analysis was focused on the 4a exon variants, comparing their properties with exons at the N-terminal (1–4) and C-terminal (9–13) was informative. As expected, the C-terminal with the MTBD was highly conserved across all vertebrates with homology originating from invertebrates (Gottschalk and Hefti, 2022). The comparison to the N-terminal exons, which contains functional domains associated with interaction of tau with other cellular components, underscores the difference between the evolution of functional domains and a strictly structural domain (Hernandez et al., 2020). For the former, there is a need to conserved sequence homology that define distinct interactions (e.g., with other proteins), while for a structural domain designed to increase the size and folding of the protein, sequence homology is not preserved. The overview relationship of the 4a and 4a-L exons among the species we analyzed is depicted in phylogenic trees (Supplementary Figures 2, 3) generated by the COBALT program following a multiple sequence alignment.

During the homology analysis, we also discovered that the “enigmatic” exon 8, whose expression remains controversial (Ruiz-Gabarre et al., 2022), can be associated with MAPT genes that express the 4a exon. A BLAST search detected exon 8 in primates with up to 100% identity, a variety of bats and carnivores including many of the cat family at 83% sequence identity. However, if we used the exon 8 sequence from bovine (Chen et al., 1994), which has only a 72% identity to human exon 8, we discovered homology with a wide range of even toed ungulates as well as carnivores (mostly the cat family). We did not, however, find that exon 8 is always present together with exon 4a variants (see exon structures in Supplementary Figure 1).

### Mechanisms

MAPT/Tau likely evolved from a prototype of MTBD sequence in invertebrates related to MAP4, following duplication into MAP2 and tau by adding N-terminal exons specific to the two new proteins (Sundermann et al., 2016). Indeed, when we searched all the way to the jawless fishes (cyclostomes) at the very beginning of the vertebrates subphylum over 500 million years ago, we found no significant homology to human tau (19%–21% sequence identity), but when analyzed for the MTBD sequence the identity was about 40% with >50% similarity. We found that large size 4a-like exons were present in early vertebrates including bony fishes, but all the way to birds they had low or no homology to the mammal or human 4a exons. They remain at low homology even when compared among the different classes (bird-reptile, 30%, bird-amphibian 20%, bird-fish 10%, reptile-amphibian, 19%, and amphibian-fish, 18%). Although we did not investigate the chondrichthyes (e.g., chimeras, skates, sharks), the presence of MAPT/MAP4/MAP2 at that evolutionary stage confirms our conclusions about the perspective about the evolution of the MAPT gene. What seems to be the case is that as the family microtubule-associated proteins evolved from a basic microtubule-binding domain in invertebrate MAPT gradually added functional exons (e.g., exons 1–4 at the N-terminal) which remained conserved. In contrast, exon 4a is extremely unconserved with no homology among different species groups (e.g., fishes, amphibians, birds, and mammals).

We therefore posit that 4a exons evolved independently in different species by exonization, where intronic sequences, which are widely diverse among species, become exons *de novo* by alternative splicing (Sorek et al., 2004). A second exonization event may have occurred later in mammal with the formation of 4a-L. Indeed, transposable elements (TE) create new sequences through exon shuffling from different genes or insertion of retrocopies within introns of existing genes (Nishihara, 2020; Almojil et al., 2021). This allows genomic sequences that did not function as exons to become coding sequences. Such events are in fact widely documented in many genomes including human, mouse, dog, and fish (Fambrini et al., 2020; Almojil et al., 2021). Exonization is a highly efficient way to add new functional modules with minimal risk for deleterious effects. Much of the observed exonizations are lineage specific. For example, 62% of new exons in human are associated with primate-specific *Alu* retroposons, and 28% of new exons in rodents are derived from rodent-specific SINE (Sorek et al., 2004). Canine-specific elements were also documented as a source for dog-specific exonizations. It therefore seems that in vertebrates, and especially mammals, the exonization mechanism is being used as a major source for accelerated, lineage-specific evolution, and may serve as a key driving force to eventual speciation.

### Summary and conclusion

Tau evolved from prototypes of MTBD sequences found in invertebrate including *C. elegans* and Drosophila (Goedert et al., 1996; Heidary and Fortini, 2001), which were originally related to MAP4 (Sundermann et al., 2016). It was followed in vertebrates by duplication into MAP2 and tau genes adding N-terminal exons specific to the 2 new proteins (Brandt et al., 2020). Indeed, when we go all the way to the jawless fishes at the very beginning of vertebrates, we find no significant homology to human tau (19%–21%), but when analyzed for the MTBD the homology was about 40% sequence identity with >50% similarity. At that point, it was not possible to identify a 4a-like exon and there was still no significant homology to the N-terminal either, both remaining at <20% sequence identity. When examining bony fishes (Table 3), we were able to identify a large 4a-like exon of 200-320aa with no significant homology to the human 4a (or any mammal 4a). There was still no homology to the N-terminal sequences either, but high homology of >50% sequence identity to the MTBD. We begin to find homology at the N-terminal sequence with amphibians (30%–40% sequence identity) increasing to 60% with reptiles and up to 70% in birds. At the same time, there was a clear 4a exon of similar size to mammals and human of about 250aa but no significant homology. The homology to the MTBD meanwhile increased to >90% sequence identity. In mammal, the N-terminal homology increased to about 80%–90% sequence identity, the MTBD close to 100%, but the 4a exons remained consistently less conserved with homology of 50%–70%, sequence identity much lower in marsupials. A different picture emerges in primate where the homology of the 4a exons variant is high with 80%–90% identity in monkeys and almost 100% in apes (Holzer et al., 2004). Assuming that the 4a is designed to change the overall 3D structure of tau without major post-translational modifications, the very high homology in primate along a span of 5–20 M years (monkeys to apes) suggests a possible selection for conservations associated with increased complexity of the nervous system. It seems that Big tau which is expressed mostly in the PNS adapted an optimized structure in primates and selective expression in CNS regions which project to the periphery. Another interesting idea is that Big tau, which can provide protection from pathological consequences has become particularly important in primates and human.

We therefore propose that at the early stages of vertebrate evolution starting with the fish taxa the evolving MAP family including MAPT remained mostly with the MTBD, and in some cases even expanded the family with zebrafish having two clusters of tau (Gottschalk and Hefti, 2022). Distinct N-terminal domains started to evolve defining exons 1–4, probably by stable exonization with amphibian, and remained highly conserved from birds to mammals. In contrast, the exonization of 4a seems to be species-specific with a narrow range of size as the principal constraint, but no pressure on sequence homology or conservation of regions associated with phosphorylation, which characterize the other regions of the tau protein. Interestingly, there may have been another exonization event to produce the 4a-L exon in mammals (mostly primates and carnivores). It is difficult to distinguish this model from a prototype of 4a whose sequence gradually changed along vertebrate evolution, but the emergence of 4a and then 4a-L makes us favor the species-specific exonization model.

Regardless of the model, the 4a exon defining Big tau appears to be present early as a very large insert that dramatically changed the size of the tau protein and likely its 3D structure. There is evidence from analysis of transcripts in Xenopus tadpoles about the early expression of the Big tau variant in amphibians. Given that tau is expressed mostly in the nervous system, it seems that in addition to LMW tau, generating multiple isoforms at the evolving N-terminal, the Big tau variant evolved with the 4a insert to generate a larger protein. It remains unknown whether the ancient Big tau had distinct distribution to peripheral systems, but analysis of the frog transcripts has shown that the XTP-1 and XTP-2 isoforms of tau were expressed in the tail, associated with peripheral neurons, while XTP-2, the shorter form expressed only in the head (Olesen et al., 2002). Although it is remarkable to have observed differential expression of tau-like isoforms in frog in the peripheral and central nervous, it is not clear from that study whether the expression was limited to neuronal cells. Consistent with the properties of tau in mammalian, another study of tau expression in Xenopus (Viereck et al., 1988) found that a HMW isoform of tau increased in the adult and stained in the cerebellum Purkinje cell bodies and parallel fibers of the molecular layer. Indeed, our search of the MAPT orthologs in Xenopus in available databases revealed that those isoforms (e.g., ENSXETT00000084149.2) contain a 4a-like exon of 226aa, but no homology to the 4a exon of mammal and human. At the same time, the MTBD had almost 80% identity. A recent study found that Big tau in Xenopus is expressed in several isoforms (with and without exons 4 and 6) suggesting that these isoforms could be used for different functions such as neurite outgrowth in embryos and neurodegeneration in metamorphosing tail spinal cord (Liu et al., 2015). The study also reported the effects of Big tau in promoting neurite outgrowth which may be consistent with its expression in mammal peripheral neurons, to support long projecting axons. Similarly, analysis of tau-like proteins (TLPs) expression in the goldfish (Liu et al., 1997) revealed that they were recognized by antibodies against the C-terminal domain of the MTBD, but not antibodies against the N-terminus. The TLPs had the typical properties of tau proteins such as neuronal specificity and structural heterogeneity. Sequence analysis of the goldfish in the Ensemble database ENSCART00000063453.1 showed that the 791aa protein contains a 4a-L size exon of 397aa similar to what we found in the carp (Table 3A). Taken together we conclude that Big tau evolved multiple independent times as a specialized isoform containing a large “inert” insert whose sequence can vary significantly across vertebrate species. In contrast, the LMW “conventional” tau has multiple isoforms and functional domains at both the N-and C-terminals and are highly regulated in the brain at the transcriptional and post-translational (mostly phosphorylation) levels (Caillet-Boudin et al., 2015). We are left wondering whether Big tau was an adaptation to a specialized tau structure by inserting 4a axon into the LMW tau or whether Big tau was the original prototype that was modified into the LMW tau without the 4a exon to address the requirements of increasing plasticity of neurons in the mammal brain (e.g., cortex, hippocampus) with the undesired consequences of a vulnerable protein that can aggregate and contribute to neurodegeneration. The fact that neurodegeneration occurs late in life may have kept such a tradeoff advantageous. Interestingly, some regions of the brain such as the cerebellum, the optic nerve and the retina, and sensory neurons that express Big tau may be less vulnerable to the type of neurodegeneration seen in tauopathies. The idea that Big tau may protect from aggregation and neurodegeneration (Fischer and Baas, 2020) is speculative at this point, but it is a testable hypothesis. For example, would the change of expression from Big tau to LMW tau (by removing the 4a exon) increase vulnerability of neurons? Another example is the visual system, where some but not all of the retinal ganglion cells express Big tau (unpublished). Does the expression of Big tau correlate with neurodegeneration, and/or their survival and regenerative potential after optic nerve crush? Ultimately, animal models of tauopathies (Mellone et al., 2013) and clinical studies will provide pathological data to resolve these important issues.

There are many remaining questions shrouded in the mysteries of Big tau concerning its evolution and function, its pattern of expression by alternative spicing of exons 2, 3, 6, 8, and 10, and whether and where Big tau with the large exon 4a-L is expressed relative to Big tau with exon 4a. Indeed, it is likely that future studies will reveal growing structural and functional complexities of Big tau with respect to multiple isoforms and the regulation of its localization (Moschner et al., 2014) in both the nervous system and other tissue (Gu et al., 1996) as well as the relationship of Big tau isoform expression with cancer (Souter and Lee, 2010). Moreover, unlocking the mysteries of Big tau may lead to a better understanding of tau as a multifunctional protein and novel strategies for treating tauopathies.

## Data availability statement

The datasets presented in this study can be found in online repositories. The names of the repository/repositories and accession number(s) can be found in the article/Supplementary material.

## Author contributions

IF designed the project, carried out the analysis, and wrote the manuscript.

## Funding

This study was funded by the William P. Snyder, III Chair Endowment to IF.

## Conflict of interest

The author declares that the research was conducted in the absence of any commercial or financial relationships that could be construed as a potential conflict of interest.

## Publisher’s note

All claims expressed in this article are solely those of the authors and do not necessarily represent those of their affiliated organizations, or those of the publisher, the editors and the reviewers. Any product that may be evaluated in this article, or claim that may be made by its manufacturer, is not guaranteed or endorsed by the publisher.

## Footnotes

^1^ https://useast.ensembl.org/Homo_sapiens/Gene/Compara_Ortholog?db=core;g=ENSG00000186868;r=17:45894527-46028334

^2^ https://www.orthodb.org/?query=716848at2759

^3^ https://www.ncbi.nlm.nih.gov/gene/101004302/
