# Supplementary material for: Evolutionary perspective of Big tau structure: 4a exon variants of MAPT
Source: Front Mol Neurosci. 2022 Dec 2;15:1019999. doi: 10.3389/fnmol.2022.1019999 (PMC9755724; doi:10.3389/fnmol.2022.1019999)
Supplement: Supplementary file 1 [file Data_Sheet_1.docx]

Supplementary Material

**Supplementary Material**

**Supplementary Fig. 1:** List of MAPT sequences used for analysis

**TABLE OF CONTENT**

**PRIMATES**

**Homo sapiens** microtubule associated protein tau (MAPT)

776aa (exon 4a)

ENST00000415613.6 MAPT-205

NCBI: NM_001123066.4, transcript variant 6, mRNA

UniProt:P10636-9

833aa (exon 4a-L)

ENST0000026-410.10 MAPT-201

NCBI: NM_001377265.1, transcript variant 9, mRNA

UniProt A0A7I2PJZ2

**Chimpanzee** (Pan troglodytes) MAPT

776 aa (exon 4a)

ENSPTRT00000094329.1

UniProt: A0A2J8J3S2

862aa (exon 4a-L)_

NCBI: XP_009430181.1, isoform X1

**Gibbon** Hylobates lar (Common gibbon) (White-handed gibbon) MAPT

776aa (exon 4a)

UniProt: Q5YCV9

Also (Northern white-cheeked gibbon) (Hylobates leucogenys)

732aa identical exon 4a

ENSNLET00000006126.2 MAPT-202

Not included in analysis

**Gorilla** (Western gorilla) MAPT

776aa (exon 4a)

UniProt: Q5YCW0

767aa (exon 4a-L)

NCBI: XP_030866931.1, isoform X1

Also, 796aa (identical exon 4a-L)

NCBI: XP_030866938.1, isoform X8

Not included in analysis

**Baboon**  (olive baboon, Papio Anubis)) MAPT

780aa (exon 4a)

ENSPANT00000017634.3 MAPT-201

UniProt: A0A096P681

852aa (exon 4a-L)

NCBI: XP_017805564.2 , isoform X1

**Marmoset** (Callithrix jacchus white-tufted-ear marmoset) MAPT

852aa (exon 4a-L)

ENSCJAT00000039196.4, MAPT-204

NCBI: XP_035156146.1, isoform X1

**Lemur** (Greater bamboo lemur, Hapalemur simus) MAPT

767aa (exon 4a)

UniProt: A0A8C9DIA7

also (Gray mouse lemur, Lemur murinus)

765aa (similar exon 4a)

ENSMICT00000068530.1 MAPT-203

UniProt: A0A8C5YBG0

Not included in analyis

**MAMMALS**

**Mouse** Mus musculus MAPT

749aa (exon 4a)

ENSMUST00000106989.3-MAPT-203

UniProt: A2A5Y6

NCBI: XP_036012272.1, isoform X2

**Rat** Rattus norvegicus MAPT

750 (775)aa (exons 4a)

ENSRNOT00000042984.6, MAPT-207

UniProt: F1LST4

Also 770aa (identical exon 4a)

NCBI: XP_008766504.1, isoform X1

Not included in analysis

**Dog** Canis lupus familiaris MAPT

869aa (exon 4a-L)

ENSCAFT00845039827.1

Also 765 (exon 4a)

NCBI: NP_001104271.2

Not included in the analysis

**Cat** Felis catus domestic cat MAPT

778aa (exon 4a)

ENSFCAT00000060163.2, MAPT-201

UniProt: A0A337S7H8

**Elephant** African savanna elephant MAPT

760aa (exon 4a)

ENSLAFT00000003277.3, MAPT-201

UniProt: G3SS28

**Opossum** Gray short-tailed opossum MAPT

860 (1051) aa (exon 4a-L)

ENSMODT00000087496.1

**VERTEBRATES NON MAMMALS**

REPTILES

**Turtle** Western painted turtle

828aa (exon 4a-L)

ENSCPBT00000045293.1 MAPT-202

**Crocodile** Australian saltwater crocodile MAPT

889aa (exon 4a-L)

ENSCPRT00005013350.1, MAPT-201

BIRDS

**Zebra Finch** MAPT

705aa (exon 4a)

ENSTGUT00000021320.1

**Golden Eagle** MAPT

836aa (exon 4a)

ENSACCT00020019941.1 (MAPT-203)

AMPHIBIANS

**Frog** tropical clawed frog, Xenopus tropical MAPT

745aa (exon 4a)

ENSXETT00000084149.2 MAPT-206

UniProt: A0A6I8RGV8

**Toad** Leishan spiny toad MAPT

760aa (exon 4a)

ENSLLET00000029521.1

FISH

**Salmon** Atlantic Salmon

731aa (exon 4a)

ENSSSAT00000242677.1, Tau-208

**Carp** Common carp

687aa (exon 4a)

ENSCCRT00000178256.1, MAPTA

814aa (exon 4a-L)

ENSCCRT00000171180.1, MAPTB-204

JAWLESS FISH

**Hagfish** Inshore hagfish MAP taub

331aa

ENSEBUT00000021644.1

**Lamprey** Sea lamprey MAP

277aa

ENSPMAT00000002181.1,

UniPort: S4RAD9

**INVERTEBRATES**

**Drosophila melanogaster** tau, isoform A

361aa

NCBI: NP_651575.1

**SEQUENCE DATABASE**

**PRIMATES**

**Homo sapiens** microtubule associated protein tau (MAPT)

776aa (exon 4a)

ENST00000415613.6 MAPT-205

NCBI: NM_001123066.4, transcript variant 6, mRNA

UniPort:P10636-9

1. MAEPRQEFEVMEDHAGTYGLGDRKDQGGYTMHQDQEGDTDAGLK
2. ESPLQTPTEDGSEEPGSETSDAKSTPTAE
3. DVTAPLVDEGAPGKQAAAQPHTEIPEGTT
4. AEEAGIGDTPSLEDEAAGHVTQ

4a)EPESGKVVQEGFLREPGPPGLSHQLMSGMPGAPLLPEGPREATRQPSGTGPEDTEGGRHAPELLKHQLLGDLHQEGPPLKGAGGKERPGSKEEVDEDRDVDESSPQDSPPSKASPAQDGRPPQTAAREATSIPGFPAEGAIPLPVDFLSKVSTEIPASEPDGPSVGRAKGQDAPLEFTFHVEITPNVQKEQAHSEEHLGRAAFPGAPGEGPEARGPSLGEDTKEADLPEPSEKQPAAAPRGKPVSRVPQLK

1. ARMVSKSKDGTGSDDKKAK
2. TSTRSSAKTLKNRPCLSPKHPTPGSSDPLIQPSSPAVCPEPPSSPKYVSSVTSRTGSSGAKEMKLK
3. GADGKTKIATPRGAAPPGQKGQANATRIPAKTPPAPKTPPSS
4. ATKQVQRRPPPAGPRSER
5. GEPPKSGDRSGYSSPGSPGTPGSRSRTPSLPTPPTREPKKVAVVRTPPKSPSSAKSRLQTAPVPMPDLKNVKSKIGSTENLKHQPGGGK
6. VQIINKKLDLSNVQSKCGSKDNIKHVPGGGS
7. VQIVYKPVDLSKVTSKCGSLGNIHHKP
8. GGGQVEVKSEKLDFKDRVQSKIGSLDNITHVPGGGNKK
9. IETHKLTFRENAKAKTDHGAEIVYKSPVVSGDTSPRHLSNVSSTGSIDMVDSPQLATLADEVSASLAKQGL

833aa (exon 4a=L)

ENST0000026-410.10 MAPT-201

NCBI: NM_001377265.1, transcript variant 9, mRNA

UniPort A0A7I2PJZ2

1. MAEPRQEFEVMEDHAGTYGLGDRKDQGGYTMHQDQEGDTDAGLK
2. ESPLQTPTEDGSEEPGSETSDAKSTPTAE

4) AEEAGIGDTPSLEDEAAGHVTQ

4a-L) EELRVPGRQRKAPERPLANEISAHVQPGPCGEASGVSGPCLGEKEPEAPVPLTASLPQHRPVCPAPPPT

GGPQEPSLEWGQKGGDWAEKGPAFPKPATTAYLHTEPESGKVVQEGFLREPGPPGLSHQLMSGMPGAPLLPEGPREATRQPSGTGPEDTEGGRHAPELLKHQLLGDLHQEGPPLKGAGGKERPGSKEEVDEDRDVD

ESSPQDSPPSKASPAQDGRPPQTAAREATSIPGFPAEGAIPLPVDFLSKVSTEIPASEPDGPSVGRAKGQDAPLEFTFHVEITPNVQKEQAHSEEHLGRAAFPGAPGEGPEARGPSLGEDTKEADLPEPSEKQPAAAPRGKPVSRVPQLK

5) ARMVSKSKDGTGSDDKKAKTSTRSSAKTLK

6)NRPCLSPKHPTPGSSDPLIQPSSPAVCPEPPSSPKYVSSVTSRTGSSGAKEMKLK

7) GADGKTKIATPRGAAPPGQKGQANATRIPAKTPPAPKTPPSS

8)

9)GEPPKSGDRSGYSSPGSPGTPGSRSRTPSLPTPPTREPKKVAVVRTPPKSPSSAKSRLQTAPVPMPDLKNVKSKIGSTENLKHQPGGGK

10) VQIINKKLDLSNVQSKCGSKDNIKHVPGGGS

11) VQIVYKPVDLSKVTSKCGSLGNIHHKP

12) GGGQVEVKSEKLDFKDRVQSKIGSLDNITHVPGGGNKK

13)IETHKLTFRENAKAKTDHGAEIVYKSPVVSGDTSPRHLSNVSSTGSIDMVDSPQLATLADEVSASLAKQGL

**Chimpanzee** (Pan troglodytes) MAPT

776 aa (exon 4a)

ENSPTRT00000094329.1

UniPort: A0A2J8J3S2

1. MAEPRQEFEVMEDHAGTYGLGDRKDQGGYTMHQDQEGDTDAGLK
2. ESPLQTPTEDGSEEPGSETSDAKSTPTAE
3. DVTAPLVDEGAPGKQAAAQPHTEIPEGTT
4. AEEAGIGDTPSLEDEAAGHVTQ

4a)EPESGKVVQEGFLREPGPPGLSHQLMSGMPGAPLLPEGPREATRQPSGTGPEDTEG

GRHAPELLKHQLLGDLHQEGPPLKGAGGKERPGSKEEVDEDRDVDESSLQDSPPSKASPA

QDGRPPQTAAREATSIPGFPAEGAIPLPVDFLSKVSTEIPASEPDGPSAGRAKGQDAHLE

FTFHVEITPNVQKEQAHSEEHLGRAAFPGAPGEGPEARGPSLGEDTKEADLPEPSEKQPA

AAPRGKPVSRVPQLK

1. ARMVSKSKDGTGSDDKKAK
2. TSTRSSAKTLKNRPCLSPKHPTPGSSDPLIQPSSPAVCPEPPSSPKYVSSVTPRTGSSGAKEMKLK
3. GADGKTKIATPRGAAPPGQKGQANATRIPAKTPPAPKTPPSS
4. ATKQVQRRPPPAGPRSER
5. GEPPKSGDRSGYSSPGSPGTPGSRSRTPSLPTPPTREPKKVAVVRTPPKSPSSAKSRLQTAPVPMPDLKNVKSKIGSTENLKHQPGGGK
6. VQIINKKLDLSNVQSKCGSKDNIKHVPGGGS
7. VQIVYKPVDLSKVTSKCGSLGNIHHKP
8. GGGQVEVKSEKLDFKDRVQSKIGSLDNITHVPGGGNKK
9. IETHKLTFRENAKAKTDHGAEIVYKSPVVSGDTSPRHLSNVSSTGSIDMVDSPQLATLADEVSASLAKQGL

862aa (exon 4a-L)_

NCBI: XP_009430181.1, isoform X1

1. MAEPRQEFEVMEDHAGTYGLGDRKDQGGYTMHQDQEGDTDAGLK
2. ESPLQTPTEDGSEEPGSETSDAKSTPTAE
3. DVTAPLVDEGAPGKQAAAQPHTEIPEGTT
4. AEEAGIGDTPSLEDEAAGHVTQ

4a-L) EELRVPGRQRKAPERPLANEISAHVQPGPCGEASGVSGPCLGEKEPEAPVLLTASLPQHRPVCPAPPP

TGGPQEPSLEWRQKGGDWAEKGPAFPKPATTAYLHTEPESGKVVQEGFLREPGPPGLSHQLMSGMPGAPLLPEGPREATRQPSGTGPEDTEGGRHAPELLKHQLLGDLHQEGPPLKGAGGKERPGSKEEVDEDRDVDESSLQDSPPSKASPAQDGRPPQTAAREATSIPGFPAEGAIPLPVDFLSKVSTEIPASEPDGPSAGRAKGQDAHLEFTFHVEITPNVQKEQAHSEEHLGRAAFPGAPGEGPEARGPSLGEDTKEADLPEPSEKQPAAAPRGKPVSRVPQK

5) ARMVSKSKDGTGSDDKKAK

6) TSTRSSAKTLKNRPCLSPKHPTPGSSDPLIQPSSPAVCPEPPSSPKYVSSVTPRTGSSGAKEMKLK

7) GADGKTKIATPRGAAPPGQKGQANATRIPAKTPPAPKTPPSS

8)

9)GEPPKSGDRSGYSSPGSPGTPGSRSRTPSLPTPPTREPKKVAVVRTPPKSPSSAKSRLQTAPVPMPDLKNVKSKIGSTENLKHQPGGGK

10) VQIINKKLDLSNVQSKCGSKDNIKHVPGGGS

11) VQIVYKPVDLSKVTSKCGSLGNIHHKP

12) GGGQVEVKSEKLDFKDRVQSKIGSLDNITHVPGGGNKK

13)IETHKLTFRENAKAKTDHGAEIVYKSPVVSGDTSPRHLSNVSSTGSIDMVDSPQLATLADEVSASLAKQGL

**Gibbon** Hylobates lar (Common gibbon) (White-handed gibbon)

776aa (exon 4a)

UniPort: Q5YCV9

1. MAEPRQEFDVMEDHAGTYGLGDRKDQGGYTMLQDQEGDTDAGLK
2. ESPLQTPAEDGSEEPGSETSDAKSTPTAE
3. DVTAPLVDEGAPXKQAAAQPHTEIPEGTT
4. AEEAGIGDTPSLEDEAAGHVTQ

4a)EPESGKVVREGFLGEPGPRSXSHQLASGMPGAPLLPEGPR

EATRQPSGTGPEDTEGGRHAPELLKHQLLGDLHQEGPPLKRAGGKERPGIKEEVDEDRDVDE

SSPQDSPPSKVSPAHDGRPPQTAAREATSIPGFPAEGAIPLPVDFLSKVSTEIPASEPDGPSAGRAEGQDAPPE

FTFHVEITPNVQKEQAHSEEHLGRAAFPGAPGEGPEAQGPSLGEDTKEADLPEPSEKQPA

AAPRGKPISRVPQLK

1. ARMVSKSKDGTGSDDKKAK
2. TSTRSSAKTLKNRPCLSPKHPTPGSSDPLIQPSSPAVCPEPPSSPKYVSSVTXRTGSSGAKEMKLK
3. GADGKTKIATPRGAAPPGQKGQANATRIPAKTPPAPKTPPSS
4. VTKQVQRRPPPAGPKSER
5. GEPPKSGDRSGYSSPGSPGTPGSRSRTPSLPTPPTREPKKVAVVRTPPKSPSSAKSRLQTAPVPMPDLKNVKSKIGSTENLKHQPGGGK
6. VQIINKKLDLSNVQSKCGSKDNIKHVPGGGS
7. VQIVYKPVDLSKVTSKCGSLGNIHHKP
8. GGGQVEVKSEKLDFKDRVQSKIGSLDNITHVPGGGNKK
9. IETHKLTFRENAKAKTDHGAEIVYKSPVVSGDTSPRHLSNVSSTGSIDMVDSPQLATLADEVSASLAKQGL

Also (Northern white-cheeked gibbon) (Hylobates leucogenys)

732aa identical exon 4a

ENSNLET00000006126.2 MAPT-202

1. MAEPRQEYDVMEDHAGTYGLGDRKDQGGYTMLQDQEGDTDAGLK
2. ESPLQTPAEDGSEEPGSETSDAKSTPTAE
3. DVTAPLVDERAPGEQAAAQPHTEIPEGTT
4. AEEAGIGDTPSLEDEAAGHVTQ

4a) EPESGKVVREGFLGEPGPPGLSHQLVSGMPGAPLLPEGPREATRQPSGTGPEDTEG

RRHAPELLKHQLLGDLHQEGPPLKGAGGKERPRSKGEVDEDRDVDESSPQDSPPSKFSPA

HDGRPPQTAAREATSIPGFPAEGAIPLPVDFLSKVSTEIPASEPDGPSAGRAEGQDAPPE

FTFHVEITPNVQKEQAHSEEHLGRAAFPGAPGEGPEARGPSLGEDTKEADLPEPSEKQPA

AAPRGKPVSRVPQLK

1. ARMVSKSKDGTGSDDKKAK
2. TSTRSSAKTLKNRPCLSPKHPTPGSSDPLIQPSSPAVCPEPPSSPKYVSSVTPRTGSSGAKEMKLK
3. GADGKTKIATPRGAAPPGQKGQANATRIPAKTPPAPKTPPSS
4. ATKQVQRRPPPAGPKSER
5. RTPPKSPSSAKSRLQTAPVPMPDLKNVKSKIGSTENLKHQPGGGK
6. VQIINKKLDLSNVQSKCGSKDNIKHVPGGGS
7. VQIVYKPVDLSKVTSKCGSLGNIHHKP
8. GGGQVEVKSEKLDFKDRVQSKIGSLDNITHVPGGGNK
9. KIETHKLTFRENAKAKTDHGAEIVYKSPVVSGDTSPRHLSNVSSTGSIDMVDSPQLATLA

DEVSASLAKQGL

**Gorilla** (Western gorilla) MAPT

776aa (exon 4a)

UniProt: Q5YCW0

1. MAEPRQEFEVMEDHAGTYGLGDRKDQGGYTMLQDQEGDTDAGLK
2. ESPLQTPTEDGSEEPGSETSDAKSTPTAE
3. DVTAPLVDEGAPGEQAAAQPHTEIPEGTT
4. AEEAGIGDTPSLEDEAAGHVTQ

4a) EPESGKVVQEGFLREPGPPGLSHQLMSGMPGAPLLPEGP

REATRQPSGTGPEDTEGGRHAPELLKHQLLGDLHQEGPPLKGAGGKERPGSKEEVDEDRDV

DESSPQDSPPSKASPAQDGRPPQTAAREATSIPGFPAKGAIHLPVDFLSKVSTEIPASEPD

GPSAGRAKGQDAPLEFTFHVEITPNVQKEQAHSEEHLGRAAFPGAPGEGPEARGPSLGEDT

KEADLPESSEKQPAAAPRGKPVSRVPQLK

1. ARMVSKSKDGTGSDDKKAK
2. TSTRSSAKTLKNRPCLSPKHPTPGSSDPLIQPSSPAVCPEPPSSPKYVSSVTPRTGSSGAKEMKLK
3. GADGKTKIATPRGAAPPGQKGQANATRIPAKTPPAPKTPPSS
4. ATKQVQRRPPPAGPRSER
5. GEPPKSGDRSGYSSPGSPGTPGSRSRTPSLPTPPTREPKKVAVVRTPPKSPSSAKSRLQTAPVPMPDLKNVKSKIGSTENLKHQPGGGK
6. VQIINKKLDLSNVQSKCGSKDNIKHVPGGGS
7. VQIVYKPVDLSKVTSKCGSLGNIHHKP
8. GGGQVEVKSEKLDFKDRVQSKIGSLDNITHVPGGGNKK
9. IETHKLTFRENAKAKTDHGAEIVYKSPVVSGDTSPRHLSNVSSTGSIDMVDSPQLATLADEVSASLAKQGL

767aa (exon 4a-L)

NCBI: XP_030866931.1, isoform X1

1. MAEPRQEFEVMEDHAGTYGLGDRKDQGGYTMLQDQEGDTDAGLK
2. ESPLQTPTEDGSEEPGSETSDAKSTPTAE
3. AEEAGIGDTPSLEDEAAGHVTQ

4a-L) EELRVPGRQRKAPERPLDNEISAHVQPGPCGEASGVSGPCLGEKE

PEAPVPLTASLPQHHPVCPAPPPTGGPQEPSLEWGQKGGDWAEKGPAFPKPATTAYLHTEPESGKVVQEG

FLREPGPPGLSHQLMSGMPGAPLLPEGPREATRQPLGTGPEDTEGGRHAPELLEHQLLGDLHQEGPPLKG

AGGKERPGSKEEVDEDRDVDESSPQDSPPSKASPAQDGRPPQTAAREATSIPGFPAKGAIHLPVDFLSKV

STEIPASEPDGPSAGRAKGQDAPLEFTFHVEITPNVQKEQAHSEEHLGRAAFPGAPGEGPEARGPSLGED

TKEADLPESSEKQPAAAPRGKPVSRVPQLK

1. ARMVSKSKDGTGSDDKKAK
2. GADGKTKIATPRGAAPPGQKGQANATRIPAKTPPAPKTPPSS
3. GEPPKSGDRSGYSSPGSPGTPGSRSRTPSLPTPPTREPKKVAVVRTPPKSPSSAKSRLQTAPVPMPDLKNVKSKIGSTENLKHQPGGGK
4. VQIINKKLDLSNVQSKCGSKDNIKHVPGGGS
5. VQIVYKPVDLSKVTSKCGSLGNIHHKP
6. GGGQVEVKSEKLDFKDRVQSKIGSLDNITHVPGGGNKK
7. IETHKLTFRENAKAKTDHGAEIVYKSPVVSGDTSPRHLSNVSSTGSIDMVDSPQLATLADEVSASLAKQGL

Also, 796aa (identical exon 4a-L)

NCBI: XP_030866938.1, isoform X8

1. MAEPRQEFEVMEDHAGTYGLGDRKDQGGYTMLQDQEGDTDAGLK
2. ESPLQTPTEDGSEEPGSETSDAKSTPTAE
3. DVTAPLVDEGAPGEQAAAQPHTEIPEGTT
4. AEEAGIGDTPSLEDEAAGHVTQ

4a-L) EELRVPGRQRKAPERPLDNEISAHVQPGPCGEASGVSGPCLGEKEPEAPVPLTASLPQHHPVCPAPPP

TGGPQEPSLEWGQKGGDWAEKGPAFPKPATTAYLHTEPESGKVVQEGFLREPGPPGLSHQLMSGMPGAPLLPEGPREATRQPLGTGPEDTEGGRHAPELLEHQLLGDLHQEGPPLKGAGGKERPGSKEEVDEDRDVDESSPQDSPPSKASPAQDGRPPQTAAREATSIPGFPAKGAIHLPVDFLSKVSTEIPASEPDGPSAGRAKGQDAPLEFFHVEITPNVQKEQAHSEEHLGRAAFPGAPGEGPEARGPSLGEDTKEADLPESSEKQPAAAPRGKPVSRVPQK

1. ARMVSKSKDGTGSDDKKAK
2. GADGKTKIATPRGAAPPGQKGQANATRIPAKTPPAPKTPPSS
3. GEPPKSGDRSGYSSPGSPGTPGSRSRTPSLPTPPTREPKKVAVVRTPPKSPSSAKSRLQTAPVPMPDLKNVKSKIGSTENLKHQPGGGKV
4. QIINKKLDLSNVQSKCGSKDNIKHVPGGGS
5. VQIVYKPVDLSKVTSKCGSLGNIHHKP
6. GGGQVEVKSEKLDFKDRVQSKIGSLDNITHVPGGGNKK
7. IETHKLTFRENAKAKTDHGAEIVYKSPVVSGDTSPRHLSNVSSTGSIDMVDSPQLATLADEVSASLAKQGL

**Baboon**  (olive baboon, Papio Anubis)) MAPT

780aa (exon 4a)

ENSPANT00000017634.3 MAPT-201

UniPort: A0A096P681

1) MAEPRQEFDVMEDHAGTYGLGDRKDQEGYTMLQDQEGDTDAGLK

2) ESPLQTPAEDGSEELGSETSDAKSTPTAE

3) DVTAPLVDERAPGEQAAAQPHMEIPEGTT

4) AEEAGIGDTPSLEDEAAGHVTQ

4a)EPESGKVVQEVFLGEPGPPGLSHQLVSSMPGAPLLPEGPREATRQPSGTGPEDTES

GHHTPELLKHQLLGDLHQEGPPLKGAGGKERLGSKEEVDEDRDVDESSPQDSPPSRVSPV

QDGQPPQTAAREATSVPGFPAEGAIALPVDFLSKVSTEIPASEPEGPSAGWAEGQDVPPE

FTFHVEITPNVQKEQAHPEEDSGRAAFPGAPGEEPEARGPSLGEDTKEADLPEPTEKQPA

AAPRGKPVSRVPQLK

5) ARMVSKSKDGTGSDDKKAK

6) TSTRSSAKTLKNRPCLSPKHPTPGSSDPLIQPSSPAVCPEPPSSPKYVSSVTPRTGSSGAKEMKLK

7) GADGKTKIATPRGAAPPGQKGQANATRIPAKTPPAPKTPPSS

8) ATKQVQRKPPPAEPKSER

9) GEPPKSGDRSGYSSPGSPGTPGSRSRTPSLPTPPAREPKKVAVVRTPPKSPSSAKSRLQTAPVPMPDLKN

VKSKIGSTENLKHQPGGGK

10) VQIINKKLDLSNVQSKCGSKDNIKHVPGGGS

11) VQIVYKPVDLSKVTSKCGSLGNIHHKP

12) GGGQVEVKSEKLDFKDRVQSKIGSLDNITHVPGGGNKK

13) IETHKLTFRENAKAKTDHGAEIVYKSPVVSGDTSPRHLSNVSSTGSIDMVDSPQLATLADEVSASLAK

QEEKERL

852aa (exon 4a-L)

NCBI: XP_017805564.2 , isoform X1

1. MAEPRQEFDVMEDHAGTYGLGDRKDQEGYTMLQDQEGDTDAGLK
2. ESPLQTPAEDGSEELGSETSDAKSTPTAE
3. AEEAGIGDTPSLEDEAAGHVTQ

4a-L) EELRVPGQQRKAPERPLANEISAHVQPGPCGEASGVSGLCLGEKEPEAPIPLTASLPQHRPICPAPPPTG

GPREPSQEWGPKGGDWAEKGPTFPKSATPAYLHTEPESGKVVQEV

FLGEPGPPGLSHQLVSSMPGAPLLPEGPREATRQPSGTGPEDTESGHHTPELLKHQLLGDLHQEGPPLKG

AGGKERLGSKEEVDEDRDVDESSPQDSPPSRVSPVQDGQPPQTAAREATSVPGFPAEGAIALPVDFLSKV

STEIPASEPEGPSAGWAEGQDVPPEFTFHVEITPNVQKEQAHPEEDSGRAAFPGAPGEEPEARGPSLGED

TKEADLPEPTEKQPAAAPRGKPVSRVPQLK

1. ARMVSKSKDGTGSDDKKAK
2. TSTRSSAKTLKNRPCLSPKHPTPGSSDPLIQPSSPAVCPEPPSSPKYVSSVTPRTGSSGAKEMKLK
3. GADGKTKIATPRGAAPPGQKGQANATRIPAKTPPAPKTPPSS
4. ATKQVQRKPPPAEPKSER
5. GEPPKSGDRSGYSSPGSPGTPGSRSRTPSLPTPPAREPKKVAVVRTPPKSPSSAKSRLQTAPVPMPDLKNVKSKIGSTENLKHQPGGGK
6. VQIINKKLDLSNVQSKCGSKDNIKHVPG
7. GGSVQIVYKPVDLSKVTSKCGSLGNIHHKPGGGQVEVKSEKLDFKDRVQSKIGSLDNITHVPGGGNKK
8. IETHKLTFRENAKAKTDHGAEIVYKSPVVSGDTSPRHLSNVSSTGSIDMVDSPQLATLADEVSASLAKQGL

**Marmoset** (Callithrix jacchus white-tufted-ear marmoset)

852aa (exon 4a-L)

ENSCJAT00000039196.4, MAPT-204

NCBI: XP_035156146.1, isoform X1

1. MAEPRQEFNVMEDHTGTYGLEDQDQEGDTDTGLK
2. ESPLQTPAEDGSEEPGSESSDAKSTPTVE
3. DVTAPLVDERAPGKQAAAQPHTEIPEGTT
4. AEEAGIGDTPTPEDQAAGHVTQ

4a-L) EELRVPGQQRKAPERPRVNELSAYVQPGPCRVAPGVSGPCLREKKSEVPVPLTASLPQHRPVCPAP

PPTGGPQEPSQEWGPKGGNWAEEGPAFLKPAAPAYLHMEPESGKMVQEGFLREPGTPSLS

HQLVSGMPGAPFLPESRREATHQPLGTGPEDTKGGCHAPELLKRQLLGDLHPEELPLKGT

GSKERPGDEKEVDEDRDVDESSPQDSPPSQVSPVRDGPPPQTFTREATSVPGFPAEGTIP

LPVDFLSKVSAETPASEPDGPSAGLAEGQDVPPEFTFHIEITANVQKEQVCSEQDSERAA

FPGAPGDGAEAQGPSLGEDTKEADLPEPSEKQPAAAPQGKHVSRVPQLK

1. ARMVSKSKDGTGGDDKKAK
2. TSTRSSAKTLKARPCLSPKHPTPGSSDPLIQPSSPAVCPEPPSSPKCVSSVTPRTGSSGAKEMRFK
3. GADGKTKIATPRGTAPPGQKGQANATRIPAKTPPAPKTPPSS
4. GETPKSGDRSGYSSPGSPGTPGSRSRTPSLPTPPTREPKKVAVVRTPPKSPSSTKSRLQTAPVPMPDLKNVKSKIGSTENLKHQPGGGK
5. VQIINKKLDLSNVQSKCGSKDNIKHVPGGGS
6. VQIVYKPVDLSKVTSKCGSLGNIHHKP
7. GGGQVEVKSEKLDFKDRVQSKIGSLDNITHVPGGGNK
8. KIETHKLTFRENAKAKTDHGAEIVYKSPVVSGDTSPRHLSNVSSTGSIDMVDSPQLATLA

DEVSASLAKQGL

**Lemur** (Greater bamboo lemur, Hapalemur simus)

767aa (exon 4a)

UNIPORT: A0A8C9DIA7

1. MAEPRQEFDVMEDHAEEAYGLGDRKDQGDYTLLQDQEGDTDHGLK
2. ESPLQNPADDGSEEAGSETSDAKSTPTAE
3. DVPAPLVAERAPGEQAAARPHTEIPEGAT
4. AEEAGIGDTPNLEDQAAGHVTQGQW

4a-L) PESIEVVQESVLGEPGRRGGETSDTGSPGPSHQQVSIMPRAPPLPEGPREAT

HRPSGTGPEDTEGSRRGSELPKHRLSGDLHRQGPLPKGAGDKERLWGEEEEDEDLDVDES

SPQASPTPDGPPFQRVSREAAGVPGFPAAGAAPLPADLLSKVSAETAASEPDGPGAQEQD

SAPEFTFHVEIKANVQKEQARSELDLATAAFPGAPGEERGAPAPSSGEDAKEADLPEPSE

RRPAAGLRGKPVSRVPQLK

1. ARMVSKGKDGTGSDDKKAK
2. TSTPSSAKTLKNRPCLSPQRPTPGSSDPLIQPSSPAVCPEPPSSPKHVSSRTGGSGAKEMKRK
3. GADGKTKIATPRGAAPPGQKGANATRIPAKTPPAPKTPPGS
4. GKENVPLNLSPLEGECPLDGYSSPGSPGTPGSRSRTPSLPTPPTREPKKVAVVRTPPKSPSSAKSRLQTAPVPMPDLKNVRSKIGSTENLKHQPGGGK
5. VQIINKKLDLSNVQSKCGSKDNIKHVPGGGS
6. VQIVYKPVDLSKVTSKCGSLGNIHHKPGG
7. GQVEVKSEKLDFKDRVQSKIGSLDNITHVPGGGNKK
8. IETHKLTFRENAKAKTDHGAEIVYKSPVVSGDTSPRHLSNVSSTGSINMVDSPQLATLADEVSASLAKQGL

also (Gray mouse lemur, Lemur murinus)

765aa (similar exon 4a)

ENSMICT00000068530.1 MAPT-203

UniPort: A0A8C5YBG0

1. MAEPRQEFDVMEDHAETYGLGDRKDQGDYTLLQDQEGDTDHGLK
2. ESSPQTPADDGSEEPGSETSDAKSTPTAE
3. DVTAPLVGERAPGEQAAAQPRTEIPEGTT
4. AEEAGIGDTPNLEDQAAGHVTQGQW

4a PGSAEVVQESVLREPGRLSHQQ

VSTMPGAPPLPKGPREATQQPSGTGPEDAEG

SRRGSELPKHQLAGDLRQEGRLPKGPGGKERPWGEEEEDEDRDVDESSPQDSPPSQASPT

PAGPPFQTASREAVGVPGFPVEGAIPLPADFLSKASADAPATEPGRPGAGPAAEEQDSAP

EFTFHVEIKANVPKEQARSERDLERAVSPGEEPEAQDPSAGEDAKEADLPEPSEKRPAAG

LRGKPVSRVPQLK

1. ARMVSKGRDGTGNDDKKAK
2. TSTPSSAKTLKTRPCLSPKRPTPGSSDPPTQPCSPAVRPEPPSCPQHASSVTARTGGAGAKETKLK
3. GADGKTKIATPRGTAPPGQKGANATRIPAKTPPAPKTPPGS
4. ARKQVQRKPPPRGQ
5. SEPAKSGDRSGYSSPGSPGTPGSRSRTPSLPTPPTREPKKVAVVRTPPKSPSSAKSRLQTAPVPMPDLKNVRSKIGSTENLKHQPGGGK
6. VQIVYKPVDLSKVTSKCGSLGNIHHKP
7. GMPSGQQGSVRWTIKVRVECPGVCCVPGGGQ
8. VEVKSEKLDFKDRVQSKIGSLDNITHVPGGGNKK
9. IETHKLTFRENAKAKTDHGAEIVYKS

PVVSGDTSPRHLSNVSSTGSINMVDSPQLATLADEVSASLAKQGL

**MAMMALS**

Mouse from e-assembly ENSMUST00000106989.3 749

NCBI Reference Sequence: XP_036012272.1

1) MADPRQEFDTMEDHAGDYTLLQDQEGDMDHGLK

2) ESPPQPPADDGAEEPGSETSDAKSTPTAE

3) DVTAPLVDERAPDKQAAAQPHTEIPEGIT

4) AEEAGIGDTPNQEDQAAGHVTQ

4a) EPEKVEIFSQSLLVEPGRREGQAPDLGTSDWTRQQVSSMSGAPLLPQGLREATCQPSGTRPEDIEKS

HPASELLRRGPPQKEG

WGQDRLGSEEEVDEDLTVDESSQDSPPSQASLTPGRAAPQAGSGSVCGETASVPGLPTEG

SVPLPADFFSKVSAETQASQPEGPGTGPMEEGHEAAPEFTFHVEIKASTPKEQDLEGATV

VGVPGEEQKAQTQGPSVGKGTKEASLQEPPGKQPAAGLPGRPVSRVPQLK

5) ARVASKDRTGNDEKKAK

6) TSTPSCAKAPSHRPCLSPTRPTLGSSDPLIKPSSPAVCPEPATSPKHVSSVTPRNGSPGTKQMKLK

7) GADGKTGAKIATPRGAASPAQKGTSNATRIPAKTTPSPKTPPGS

8)

9) GEPPKSGERSGYSSPGSPGTPGSRSRTPSLPTPPTREPKKVAVVRTPPKSPSASKSRLQTAPVPMPDLKN

VRSKIGSTENLKHQPGGGK

10) VQIINKKLDLSNVQSKCGSKDNIKHVPGGGS

11) VQIVYKPVDLSKVTSKCGSLGNIHHKP

12) GGGQVEVKSEKLDFKDRVQSKIGSLDNITHVPGGGNKK

13) IETHKLTFRENAKAKTDHGAEIVYKSPVVSGDTSPRHLSNVSSTGSIDMVDSPQLATLADEVSASLA

KQGL

**Rat** Rattus norvegicus

750 (775)aa (exons 4a)

ENSRNOT00000042984.6, MAPT-207

UniPort: F1LST4

1) MAEPRQEFDTMEDQAGDYTMLQDQEGDMDHGLK

2) ESPPQPPADDGSEEPGSETSDAKSTPTAE

3) DVTAPLVEERAPDKQATAQSHTEIPEGTT

4) AEEAGIGDTPNMEDQAAGHVTQ

4a) EPQKVEIFSQSLLVEPGRREGQAPDSGISDWTHQQVPSMSGAPLPPQGLREATHQPLGTRPEDVERS

HPASELLWQESPQKEAWGKDRLGSEEEVDEDITMDESSQESPPSQASLAPGTATPQARSV

SASGVSGETTSIPGFPAEGSIPLPADFFSKVSAETQASPPEGPGTGPSEEGHEAAPEFTF

HVEIKASAPKEQDLEGATVVGAPAEEQKARGPSVGKGTKEASLLEPTDKQPAAGLPGRPV

SRVPQLK

5) ARVAGVSKDRTGNDEKKAK

6) TSTPSCAKTPSNRPCLSPTRPTPGSSDPLIKPSSPAVCPEPATSPKYVSSVTPRNGSPGTKQMKLK

7) GADGKTGAKIATPRGAATPGQKGTSNATRIPAKTTPSPKTPPGS

8)

9) GEPPKSGERSGYSSPGSPGTPGSRSRTPSLPTPPTREPKKVAVVRTPPKSPSASKSRLQTAPVPMPDLKN

VRSKIGSTENLKHQPGGGK

10) VQIINKKLDLSNVQSKCGSKDNIKHVPGGGS

11) VQIVYKPVDLSKVTSKCGSLGNIHHKP

12) GGGQVEVKSEKLDFKDRVQSKIGSLDNITHVPGGGNKK

13) IETHKLTFRENAKAKTDHGAEIVYKSPVVSGDTSPRHLSNVSSTGSIDMVDSPQLATLADEVSASLAKQ

(GKPVLLSSEVWNYSHDFGHHTDLGL)

Also 770aa (identical exon 4a)

NCBI: XP_008766504.1, isoform X1

1. MAEPRQEFDTMEDQAGDYTMLQDQEGDMDHGLK
2. ESPPQPPADDGSEEPGSETSDAKSTPTAE
3. DVTAPLVEERAPDKQATAQSHTEIPEGTT
4. AEEAGIGDTPNMEDQAAGHVTQ

4a) EPQKVEIFSQSLLVEPGRREGQAPDSGISDWTHQQVPSMSGAPLPPQGLREATHQPLGTRPEDVERSHPA

SELLWQESPQKEAWGKDRLGSEEEVDEDITMDESSQESPPSQASLAPGTATPQARSVSASGVSGETTSIPGFPAEGSIPLPADFFSKVSAETQASPPEGPGTGPSEEGHEAAPEFTFHVEIKASAPKEQDLEGATVVGAPAEEQKARGPSVGKGTKEASLLEPTDKQPAAGLPGRPVSRVPQLK

1. ARVAGVSKDRTGNDEKKAK
2. TSTPSCAKTPSNRPCLSPTRPTPGSSDPLIKPSSPAVCPEPATSPKYVSSVTPRNGSPGTKQMKLK
3. GADGKTGAKIATPRGAATPGQKGTSNATRIPAKTTPSPKTPPGS
4. ASKQPQRKLPPAGAKTER
5. GEPPKSGERSGYSSPGSPGTPGSRSRTPSLPTPPTREPKKVAVVRTPPKSPSASKSRLQTAPVPMPDLKNVRSKIGSTENLKHQPGGGK
6. VQIINKKLDLSNVQSKCGSKDNIKHVP
7. GGGSVQIVYKPVDLSKVTSKCGSLGNIHHKPGGGQVEVKSEKLDFKDRVQSKIGSLDNITHVPGGGNK
8. IETHKLTFRENAKAKTDHGAEIVYKSPVVSGDTSPRHLSNVSSTGSIDMVDSPQLATLADEVSASLAKQ

GL

**Dog** Canis lupus familiaris

869aa (exon 4a-L)

ENSCAFT00845039827.1

1) MAEPRQEFTVMEDHAGTYGKDLPSQGGYTLLQDHEGDVDHGLK

2) ESPLQTPADDGSEEPGSETSDAKSTPTAE

3) DVTAPLVDEGTPGEQAAAQPPMEIPEGAT

4) AEEAGIGDTPNLEDQAAGHVTQ

4a-L) EELRVPGQQREVPERPLANGLSSQDQPEHGREASGVSLLPLGEKRPEAPVLVLSCQS

QHHPICQPPAPTGGPRDSHQEWGLKPGDGITGGLAFPKPTSPGCLQT

ESGSDKAVQESLLGEPGRRRGEAADPGTPGQPHQHVSYGPREATQQPSGTEPEDTEGSRHGLELLEHQLLGDLCQEGPPLKGDHDKERRGGKDFDEDRDVDESSPQDSPPSQVSPGPQSSPTRGGAPPDTASR

GAPGFPGFSAEGAIRLPVDFLSNVSTEIEASEPPGPGAGPTVEGQATPPEFTFHVEIKAN

VQKEQGHSEADLEGAALPGPPGEEREPQGPSEGEDTKKTDLPEPSEKQPTAVLPEKPVSR

VPQLK

5) ARMVSKGKDGTGTDDKKAK

6) TSTPSSAKTLKNRPCLSPTRPTPGSSDPLIKPSSPAVCPESSSSPKHVSSVTPRTGSSGAKEMKAK

7) GADGKTGTKIATPRGATPTGQKGQANATRIPAKTTPSPKTPPG

8)

9) GESGKSGDRSGYSSPGSPGTPGSRSRTPSLPTPPTREPKKVAVVRTPPKSPSAAKSRLQTAPVPMPDLKNV

RSKIGSTENLKHQPGGGK

10) VQIINKKLDLSNVQSKCGSKDNIKHVPGGGS

11) VQIVYKPVDLSKVTSKCGSLGNIHHKP

12) GGGQVEVKSEKLDFKDRVQSKIGSLDNITHVPGGGNKK

13) IETHKLTFRENAKAKTDHGAEIVYKSPVVSGDTSPRHLSNVSSTGSIDMVDSPQLATLADEVSASLAK

QGL

Also 765 (exon 4a)

NCBI: NP_001104271.2

MAEPRQEFTVMEDHAGTYGKDLPSQGGYTLLQDHEGDVDHGLK

1. ESPLQTPADDGSEEPGSETSDAKSTPTAE
2. DVTAPLVDEGTPGEQAAAQPPMEIPEGAT
3. AEEAGIGDTPNLEDQAAGHVTQ

4a) ESGSDKAVQESLLGEPGRRRGEAADPGTPGQPHQHVSYGPREATQQPSGTEPEDTEGSRHGLELLEHQL

LGDLCQEGPPLKGDHDKERRGGKDFDEDRDVDESSPQDSPPSQVSPGPQSSPTRGGAPPDTASRGAPGFPGFSAEGAIRLPVDFLSNVSTEIEASEPPGPGAGPTVEGQATPPEFTFHVEIKANVQKEQGHSEADLEGAALPGPPGEEREPQGPSEGEDTKKTDLPEPSEKQPTAVLPEKPVSRVPQLK

1. ARMVSKGKDGTGTDDKKAK
2. TSTPSSAKTLKNRPCLSPTRPTPGSSDPLIKPSSPAVCPESSSSPKHVSSVTPRTGSSGAKEMKAK
3. GADGKTGTKIATPRGATPTGQKGQANATRIPAKTTPSPKTPPG
4. GESGKSGDRSGYSSPGSPGTPGSRSRTPSLPTPPTREPKKVAVVRTPPKSPSAAKSRLQTAPVPMPDLKNVRSKIGSTENLKHQPGGGK
5. VQIINKKLDLSNVQSKCGSKDNIKHVPGGGS
6. VQIVYKPVDLSKVTSKCGSLGNIHHKP
7. GGGQVEVKSEKLDFKDRVQSKIGSLDNITHVPGGGNKK
8. IETHKLTFRENAKAKTDHGAEIVYKSPVVSGDTSPRHLSNVSSTGSIDMVDSPQLATLADEVSASLAKQGL

**Cat** Felis catus domestic cat

778aa (exon 4a)

ENSFCAT00000060163.2, MAPT-201

UniPort: A0A337S7H8

1) MAEPRQDFTVMDDHAGTYGTGERKDLPSQGSYTLMQDHEGDVDQGLK

2) ESPLQTPADDGSEEPGSETSDAKSTPTAE

3) DATAPLVDEGAPGEQAAAQPHTEIPEGTT

4)AEEAGIGDTPNLEDQAAGHVTQ

4a) EPGSVKVVQEILLGEP

GTPGLPRQHAPGAPILPHGPREATHQPSGTEPEDTEGSRRDTELLEYQLVGDLRQEGPPLKGDHGKERRGGKDVDEDRDVDESSPQDSPRSHV

SPVRGGPPPQTVSRGATGLSGFTAEGAIRLPVDFLSQVSTEIEASEPPRPGAGPTAEGQDTPPG

FTFHVEIKANVQKEQARSEVDLEGAALPGPPGEEQEPQGPSEGEDTKKSDLPEPSEKQPA

AVLPGKPISRVPQLK

5) ARMVSKGRDGTGADDKKAK

6) TSTPSSAKTLKNRPCLSPKRPTPGSSDPLIKPSSPAVCPESSSSPKHVSSVTPRTGSSGAKEMKVK

7) GADGKAGTKIATPRGAAPPGQKGQANATRIPAKTTPSPKTPPGT

8) ATKQVQRKPPPAGAKSER

9) GDSGKSGDRSGYSSPGSPGTPGSRSRTPSLPTPPTREPKKVAVVRTPPKSPSSAKSRLQTAPVPMPDLKNV

RSKIGSTENLKHQPGGGK

10) VQIINKKLDLSNVQSKCGSKDNIKHVPGGGS

11) VQIVYKPVDLSKVTSKCGSLGNIHHKP

12) GGGQVEVKSEKLDFKDRVQSKIGSLDNITHVPGGGNKK

13) IETHKLTFRENAKAKTDHGAEIVYKSPVVSGDTSPRHLSNVSSTGSIDMVDSPQLATLADEVSASLAK

QGL

**Elephant** African savanna elephant

760aa (exon 4a)

ENSLAFT00000003277.3, MAPT-201

UniPort: G3SS28

1) MAEPRQEFNVMEDHAETCYALLQDHEGDADRGLK

2) ESPLQTPADDGSEEAGSEASAKSTPTAE

3) DVTAPLVDETAPGEQPHTEIPEGTT

4) AEEAGIGDTPNLEDQAAGHVTQ

4a) DLPMEPKSVKVVQEGVLGEPGHHGGQATHPGTLGLAQQLASNIPGASILPEGPRGAHQPGTEPEDTEG

SHRGSELLKCQLLEDLPQGKEKLGSEKEVDEDRDIDESSPQDSPPSQISPHPQISQTPAGPLP

QTVSRDATGIPGLPAEGAIPHPVGFLPKVSTETQVSEPDVPSAGSAGEVAEGHEAPAEFM

FQVEIKANMQKERECSQLDLEETALPGSPGEEQEARGPSVREDTQEADLPERSKKQPAAG

LSGRPVSRVPQLK

5) ARMVSKSKDGTGSDDKRAK

6) TSTPSSARTLKNRPCLSLKRPTPGSSDPLIKPSSPAVCPEPSSSPKHVSSVTPRTGSSGAKETKLK

7) GADKTGTKISTPRGAAPPGQKGAANATRIPAKTTSTPKTPPST

8)

9) GDSGKSGDRSGYSSPGSPGTPGSRSRTPSLPTPPAREPKKVAVVRTPPKSPSSAKSRLQTAPVPMPDLK

NVKSKIGSTENLKHQPGGGK

10) LSKVQIINKKLDLSNVQSKCGSKDNIKHVPGGGSV

11) QIVYKPVDLSKVTSKCGSLGNIHHKP

12) GGGQVEVKSEKLDFKDRVQSKIGSLDNITHVPGGGNKK

13) IETHKLTFRENAKAKTDHGAEIVYKSPVVSGDTSPRHLSNVSSTGSINMVDSPQLSTLAEEVSASLAK

QGL

**Opossum** Gray short-tailed opossum

860 (1051)aa (exon 4a-L)

ENSMODT00000087496.1

(MRSGASVRAPVSAGESWVGEGGAGVEIQGGRGGEERTTGGKGKNPSGRGEGECEVPCSRP

LEDSLWRPEEKTAVVKEGCLRRGDALALALALTLRLSTCCLIWASAPALVLPLLLLLLRL

LQGLVLNYTALEEAPWSRTLLLSCARPLSLIASHLRPPPTVTRLYSGQHHLNHLHSPHSP

HTGQTDQSLSR)

1) MAEQRSSYNMMEDHSGNQQIHSGEPFQIGGERKDLASQGGYTLLQDNED

DSGHGLK

2) GYSQHTPADDGSDEQVSETSAKSTPTTE

3) DVTAPLVDEQEHEDQPAAQVEIPEGTT

4) AEEAGIGDTPNLEDQAAGDVSQ

4a-L)EELRVSAECQREESEILDFSANEIKSQMPQVSVEETFQKPQTPLRKKEPEMTVPLASQDNSVSSEAPSL

EEDMKETCPALRQINGDRTEQSLPSLKPSVLESSKPSGYDHFRVGVEGHEVYGSFLSEPGHYGAEAIGQDAINMPGERISHMSAESIQSRSPGDSPLVLPVKERAGVREDDEDRDIDESSLQDSPLSSQPSPRTESLQPQEVSSPSVASKETVAISGLPVEHHIKIPLPDDFLPKIPSEMPATESKPPELSQLGTEGSDELSSFESS

ESVFQVEIKANIPKEQEFSELGPEDTVSPEDTMSGEPGSPSASVEKDAKIPEKHSLRSPS

GKPVSRVPQLK

5)ARKSSVAGSDEKKVK

6)PSTPSSAQTFLKGRPTISPKRPSPVSSDPSITPSSPAVSSVPGSSPKHISSVISHTGSSGTKAMKSK

RAESKSGIKMATHRAVPSGHKGSANATRIPAKASGPKTPPSA

9)GESTKSGERSGYSSPGSPGTPGSRSRTPSLPTPPTREPKKVAVVRTPPKSPSSTKSRLQTSAVPMPDLKNVRSKIGSTENLKHQPGGGK

10)VQIFNKKLDLSNVQSKCGSKDNIKHVPGGGS

11)VQIVYKPVDLSKVTSKCGSLGNIHHKP

12) GGGQVEVKSEKLDFKEKVQSKIGSLDNITHVPGGGNKK

13) IESHKLTFRENAKAKTDHGAEIVYKSPTMSGDTSPRHLSNVSSTGSINMVDSPQLATLADEVSASLAK

QGL

**VERTEBRATES NON MAMMALS**

REPTILES

**Turtle** Western painted turtle

828aa (exon 4a-L)

ENSCPBT00000045293.1 MAPT-202

1. MAEQRQDFNMMEDHSMSQAKQIPS
2. GYPLQIPVDDGSDEPTSETSDAKSTPTMED
3. ATAPLVEERAHEDRIAARQHVEIPEGTT
4. AEEAGVGATPNLEDQAAGDGAQ

4a-L) EELSSPRLWESVEPGI

QEHVASEIQPEKQAGEVLPATVLPLREMRTGEQAAAIPARAEATIPVPPGTYEDFRAFEE

SQEAVSRTELWDRGGKEDVGVGKALADEAGHDVCTEEFATSAVSEFSQGAPSAGMFYHEK

LAAPFEKWHPGIDDVGYEPVDNSHIQDRPYPLYSRGKLKEEAAGHEKDEDRDIDETILQD

SPSALGPQDSLLLEAAEEAVEVHGFPAEGRPKDNLAEISREIPVTERETHKAGQILDERR

QWLSGKGYDDVTKMEPFESMYQVEVEANVIREGEISGSPLGATKFPDRLNKDVADRSVSL

QQEMGGMVQAMEKEESPKKKPATRMSEKQVSRVPHLK

1. ARIDSKDKDGTDTEEKKPK
2. TSTPSSANPLKDRSSITPQRPSSVSTTPLKNPSSPAESSVPASTPKRVSSITSRPASTGRKETK

PK

1. GPEMKSGMKMAAPRSATQTQKSPANATRIPAKTPTAPKTPPNA
2. AGRKEQRKPPTPAAKSEK
3. GEPAKSGDRSGYSSPGSPGTPGSRSRTPSLPTPPNREPKKVAVVRTPPKSPASAKSR
4. LQTSTAPMPDLKNVRSKIGSTENLKHQPGGG
5. KVQIVYKPVDLSHVTSKCGSLGNIHHKPG
6. GGQVEVKSEKLDFKEKVQSKIGSLDNITHVPGGGNKK
7. IESHKLTFRENAKAKTDHGAEIIYKSPTVSGDASPRRLSNVSSTGSINMVDSPQLATLADEVSASLAKQGL

**Crocodile** Australian saltwater crocodile

889aa (exon 4a-L)

ENSCPRT00005013350.1, MAPT-201

(MQGAVSSREEGVHENFGNK)

1) MAEPRQDFSMMDDHSLSQDKQISS

2) GYPLQIPVDDGSDEPVSETSDAKSTPTTE

3) DATAPLVEEGEHEDGTGAQHHVEIPEGTT

4) AEEAGVGATPNLEDQAAGDVAE

4a-L) EELSSPKLEEGVEPGLQEHLADEIKKEIQPGKQAEKVLKELHPLLRGRPAGEQAAAA

PTRIEVRIPGLTDVSKDFTTLEESEDVVSRSLFNEPWDIGGKEGIAVDKAPLAESAHRVP

FGGFVQAAAVPELLSTQGGEWSSPEELIAGPEKWLPGVDVVGYDQVVDSGLVRDGLSCLH

ARGKIKEDAVGQEKDKDHDIDETLQQDSLPSPGQKVSPMHEACIYPGASKKALEAHVFPV

ESIPRGDLAAAYREAVSVEHKASLAHDEDNQSASEGNHDVTKLKPSESIYQVEVEAEACA

LGEGEISQLQQEAAKLSNSLQTDVDSQHVPLAEEAGSIFETVGKQTPRKRPAAHVSEKPV

SRVPLLK

5) ARVDGKDKDGTAEQKKPK

6) TSTPSSASTLKDRPSITPKRPCSVSTIPLKIPSSPAESSVPASSPKQVSSVTSRLTSTGMKETKPK

7) GLEMKSGMKMPTPKSSVAQAQRSPANASRIPAKTPTAPKTPPTT

8) AGKKEQKKPPSTAAKSDK

9) GEQPKSGDRSGYSSPGSPGTPGSRSRT

9) PSLPTPPAREPKKVAVVRTPPKSPSSAKSRMQPSAVPMPDLKNIKSKIGSTENLKHQPGGGK

10) VQIVNKKLDLGSVQARCGSKDNIKHVPGGGS

11) VQIVYKPVDLSHVTSKCGSLGNIHHKP

12) GGGQVEVKSEKLDFKDKVHSKIGSLDNITHVPGGGNKK

13) IETHKLTFRENAKAKTDHGAEIVYKSPTISGDASPRRLSNVSSSGSINMVDSPQLSTLADEVSASLAKQ

GL

BIRDS

**Zebra Finch**

705aa (exon 4a)

ENSTGUT00000021320.1

1. MEDHAPGQEKHFSP
2. GYPLQIPVDDGSDEPVSETSDAKSTPTTE
3. DATAPLVEEGDHEDQGGAEQHGEIPEGTT
4. AEEAGVGATPNLEDHAAGDATQ

4a) GEPSSPKLQPGPRERVGEAVKSASQPPEQGLGPQQPPLSRETKAPAAAPTRIEVTIPIPLDMYQGSEGSG

ELWDQGGTEGLARAGGTGGHKDGPSPLCARATIKEDSGGRERDEDRDIDETSGQGLPSLVDQCVSLAPEGSCPAAAQEAREEYDGENKSKGVLRDTPGEALLVEAESHKAGEDQEEKRELLEGEGGPDSALSEP

SGSVSLKEAEPREGEDSGPVLETAKLPAEGEDGVKKVDEDAPVGEAVPDAGGRRTPRRKP

GGLAADKASRVPLLK

1. GRVDKEGTEADEKKPK
2. GPEARGGSKTGTARAGQAQRNSTNATRIPAKTPTAPKTPPSS
3. GRKEQKKPPPAAAKTEK
4. GEQPKSGDRSGYSSPGSPGTPGSRSRTPSLPTPPAREPKKVAVVRTPPKSPASAKTRVQPSAAPMPDLKNVKSKIGSTDNLKHQPGGGK
5. VQIINKKLDFSSVQSKCGSKDNIKHIPGGGS
6. VQIVYKPVDLSHVTSKCGSLGNIHHKP
7. GGGQVEVKSEKLDFKDKVQSKIGSLDNISHVPGGGNKK
8. IETHKLTFRENAKAKTDHGAEIVYKSPTISGDASPRRLSNVSSSGSINLVDSPQLATLADEVSASLAKQGL

**Golden Eagle**

836aa (exon 4a)

ENSACCT00020019941.1 (MAPT-203)

1. MAEQRQDVTVMEDHAAGQEKHIPS
2. GYPLQIPVDDGSDEPVSETSDAKSTPTTE
3. DATAPLVEEGDHEDQGGVEQHGEIPEGTT
4. AEEAGIGATPNLEDHAAGDAAQ

4a) GEPSSPKLQPGPQERV

GDAIKRESQPTKQVAGVLQQPLLSHETKATTAAPTRIEVTIPIPLDMYQDSRASEGNNEL

WDHQGREGIGVDPALGTELGRDVRTEGLVGAGGTDDSHIKDGPSPLYTRAPLKEDASGRE

RDEDRDIDETSEQDLLSLVGKHVSSEPEMGLCPATAKKALEEYAFEENESKDVLRDIPRK

AILVETESHKAGEDQEERRQPLKGEEDTHVTPPEPSEIISEKEAEPREGEDSRPLLETAK

LPVELKDDMEDKDGPLEEAVPDTGGRRTPKKKPCAHVADKAVSRVPLLK

1. GRIDSKDKEGTEAEEKKPK
2. KSSPSTAKPPGDRPSIPPQRHTSSSTTPSKTPTSPASTSKRVSSVTSRPGSTGMQETKAK
3. GPEMRGGTKTATPRSAAGQAQRNSTNATRIPAKTPTAPKTPPSS
4. GRKEQKKPPPAAAKSEK
5. GEQPKSGDRSGYSSPGSPGTPGSRSRTPSLPTPPAREPKKVAVVRTPPKSPASAKSRIQPSAAPMPDLKNVKSKIGSTENLKHQPGGGK
6. VQIINKKLDFSSVQSKCGSKDNIKHIPGGGS
7. VQIVNKKLDFSSVQSRCGSKDNIKHIPGGGS
8. VQIVYKPVDLSHVTSKCGSLGNIHHKP
9. GGGQVEVKSEKLDFKDKVQSKIGSLDNISHVPGGGNKK
10. IETHKLTFRENAKAKTDHGAEIVYKSPTISGDASPRRLSNVSSTGSINMVDSPQLATLADEVSASLAKQGL

AMPHIBIANS

**Frog** tropical clawed frog, Xenopus tropical

745aa (exon 4a)

ENSXETT00000084149.2 MAPT-206

UniPort: A0A6I8RGV8

1. MLLSLPGESYLVLPSSGKEGVIKERERQRR
2. GSSRGRMADHYQDYDSVGDHTRDGSAQQIYTGGSGDHILESSQESVIRLPTAAHGD
3. DAASVQEDLANGEAVRSQGHAGIPEGTT
4. AEEAGVGHTPSQQDRAA

4a) EEIALLAAAGQEEEYEMDTMEETLKITAKDQTHAENYGITGDVDGESQN

DETALSSGMVESAVEEDYYKETNGKEVNLEICEDDTEGWEEQIDEGIIMQDSVAPPKGGE

QELSSVEQPQTNGTGAEHIFLEDNQHKKDTEEPFMAIPANSFPVGQIRPRASVSVYQVEI

DANIPIDSKEAPCEDVGIPGGTKVDTERATEETLKSPRKRMPAHGSGIPVSRVPVPK

1. AHEQEKHETDSQEKGAQ
2. ISTKHPPAKNAKSRLYTVPQKSPSITSKSPSSPAASSVRSIQRTSLGAVGQPGVTARLHRAK
3. EAGGPESAKSQLSASRNATSASRIPAKTSSIPKTPPS
4. AVRRDQRKPPPSGAKPDR
5. AESPKSGERSGYSSPGSPGTPTGRSSSQTPPTREPKKIAVIRTPPKSPASAKSRLQPVTSPAAMPDLKNVRSKIGSIDNIRHQPGGGK
6. VQIVHKKIDLSSVQSKCGSKDNLKHMPGGGT
7. IQITHKPIDLTHITSKCGSFGNIHHRP
8. GGGNVEVKSEKLEFDKIQSKIGSLDNITHTPGGGAKK
9. IESHKLMFRENAKAKTDHGAEIVYKSPGQSGETSPRRLSNVSSSGSINMTDSPQLSTLADQVSASLAKQGL

**Toad** Leishan spiny toad

760aa (exon 4a)

ENSLLET00000029521.1

1. MSEQYQDYDSLGDQVGDGSPDQLYSGVTGSDGTGHIQDGPQESVIRLPRVSQHGD
2. ?
3. DDEDVLKDLANGEAAREQGHLSIPEGTT
4. AEEAGVGNTPNQDRQAADNALK

4a) EEIKSPSTSEKEEIDESCDTIPQMEPPQETDITKASLESHVDTVEGGPLSSQDTCPEDLGESSEEIDADISP

NWKVNQLAKPVELQEEVHVSSEEHNGKQYHEDTSEDNDGEWGGETAVSDELTPPIEALLVDRT

KHFQPREDSYTNGTESEETPVENHDKLLDTLVFTPNDQEEVIEQPFVEIAAESFSVEAPL

PRPRPSVSVYQVEVDANKPINGEVKPSDVLDMQGGDTEEHIHTVSEVKETPRKRASGIPV

SRAPLPK

1. AHGEEKHETDSQEKGAQ
2. TSSPSPAKHSNNRTSTIPKKSPSTSTLTQKKSSSSTGPPIRSPAQRTTPVTSRLSGTTTCSYKAK
3. DGGLESTAVKQVASRSPANVSRIPSKTPTVPKTPPS
4. AVRRDQRKPPASMGKTERESPKSGERSGYSSPGSPGTPTNRSRTPSTQTPPTREP
5. KKVAVIRTPPKSPASGKSRLQPVPSSAPLPDLKNVRSKIGSIDNIRHQPGGGK
6. VQIVHKKVDLSNIQSKCGSKDNLKHAPGGGA
7. VQITHKPVDLKHVTSKCGSMANIHHKP
8. GGGNVEVKSEKLDFKEKVQSKIGSLDNVTHTPGGGTKK
9. IESHKLNFRENAKAKTDHGAEIVYKSPNPSGDTSPRRLSNVSSSGSINMADSPQLSMLADAVSASLAKQGL

FISH (note that here the exons were designated with the original alternating black/blue colors of the ENSEML protein database with first amino acid in red, and that for jawless fish there was not a specific designation of exons).

**Salmon** Atlantic Salmon

731aa (exon 4a)

ENSSSAT00000242677.1, Tau-208

1. MDQHQDYMNNAPNTYSYNSGDTMSASLAGMTINDLHHQENGVQLGHRSPGDGPMK
2. VEPEEATLEDRVAEPQSELSELESNTCDEEVQLSASCEEEVQP
3. VEPEEARPKDRVTEPQSELSELESIPCDEEVHL
4. ASGGTAST

4a) EEPGVGILSGFVLVPPSGSEAAEDTEIGAGLGFKTLGAFCS

LDSEAADSEGAQGGRDFGKKLSNSLSTSGDHTQGSEGRGDLPSTRGVSPDSQRAVSLDTP

ECSSGQSDPLGTSLVRSTAFEDLTSVEENRLWVDEDQGLRISKEAKKHGLAFDYAESLQQ

AAAASNTGSKQFVPGSPDSFPVHSPSSSHFFLENSVPVDSSPRHYSEHLQEVHDAVSSAL

KDTAHFDVDNQLERQVEPQVNGNMVSSDSDKHLTTVLSDIGVINPVAELERASYMSIPDF

VEDPKMRDVVQSTKTTSELQKTLQSSPARKSLVPVAIYK

1. AQAKIENDNADKK
2. TLGGARPQAPGTKIPAKTPAP
3. ASGKNK
4. DNTSGQSSPGTPKSPSSKTLPGKPLAVATNQVKKVAVVRTPPKSPGSLKSRAPAPLTAAAPLPDLKNVRSKIGSTDNIKHQPGGGR
5. VQILEKKLDVSNVQARCGSKANLNHTPGGGR
6. VQIVHKKIDLSNVKSKCGSTANIHHKP
7. GGGNVEIKSEKLDFKVQSKIGSMDNIGHVAGGGQRR
8. KEKGKEAGDSPSNKGTYGPSPADTPPQSPQPLPSAPITPILMNPLIKIEDSN

**Carp** Common carp

687aa (exon 4a)

ENSCCRT00000178256.1, MAPTA

1. MDASTVVDSPKSY
2. VSSFGLAMDQQHDLLSSSANSHTAHYNSGDTMATSLSGMTINDHHHGNQFHNENGIAVGLVRPGDCPMK
3. EEFSEEQVDDERSNAEGTSSAEEKQCESPQDSKQEEKE
4. DQCQPVVEMAS
5. AGGTAPA

4a) ALAARFSSDGYDQHSDWSLVDPQRILEQDEDLLRSSWSEQPV

HSLYRRTAVSPDLTRSLSLENEPPVTFLVRSTALEDLTSVRERTVEARAPYSETLQEEEE

LSFYGIESHVEEETLPVRASRAQTTLAEYAPSETTEHSVSKTLPHEHMEATGEVQEKVNA

TLSGVDHSKDDGKTEATPPSTSTIGSPKGVHISPARKSLVPVAQFK

1. AQSKTNGDAEKRTP
2. KNSKVRPSSHKTPSSIPKKTSSSRSPSVSSTSSGPKESRPR
3. APSSSRPHAAGTKIPAMTT
4. AAKNGK
5. DSPKTPETSGHSSPGTPKSPASKAAGGKPPSTGNEIKKVAVIRSTPKSPKNRSP
6. TSLSAAAPLPDLKNVRSKIGSTDNLKHQPGGGR
7. VQILDQKVDFSNVQSKCGSKANLKYVPGGGN
8. VQILDQKLDLSSVQSRCGSKDNIKHLPGGGK
9. VQILHKKIDLSNVQAKCGSKDNLHHKP
10. GGGNIEIRSEKLEFKAQSKVGSMDNIKHTPGGGSRR
11. REKGRGADTPRDEGFVTPDPSDTLTLSSASMSPEPILLSNPQIKIEDSN

814aa (exon 4a-L)

ENSCCRT00000171180.1, MAPTB-204

1. MDHQDHMNSGQVGDSQHSPGNNIASGVANMTISDGHQQDMKNGTAAHMGRADGPVK
2. EDTHEPTPEVTTQKPVLEDSEPKPSFDKDVPL
3. EDLTPGSLPESGRSSAASVDGEGERENGEKES

4a-L)SVSPRESPMSPHPLLTTLADDLGSGITGFDSQMKRSPSEKMTDHHSTSGSEEEEKEENEM

EKSERRSGSPSVEEAPRSAINIKEDKEEDKEAVSDEEEEETGISLVPSSVTPDLTVQKDD

SIDDERETPSHLPMTPEEAKKHGLSFDYTEPQDPSRQGGPVGWECSSDKTPPESKSPDSC

RADPGSPFSPSAPAEPTQEESSLTDLQTDAQEEEEEVEVPEPEQVEEATTVPPSFQEVAA

PKIQPKAEECREPEEEKEIKQEKYLETSDDDLIDTEKVQPVATPEPVAIPEPVAKTEPEA

KDAVKPSAQVMPTKAPSKASPIKESPAKKTKKPVATVAATPSPKSAPKLQKTPSKDAAPA

RKSSVPSK

1. AKAGAGATPEKK
2. IPTTTPHAKSRPTSAPQRGSSVSGIPSKNPSVSSSTPPCRFSSPGSANSVKRSKSAGARESRAT
3. AGDVKTKTAGAKPQGVGIKIP
4. AASRMEQRKAGPGSIER
5. ADSPKTPDRSGCSSPASRSSTPGQQVKKVAVVRTPPKSPGSLRSRTPIAPVAPLPDLK
6. NIKSKIGSTENIKYQPGGGK
7. VQIVHKKIDLSNVQSKCGSKVNIHHKP
8. GGGNVEIKSEKLDFKGQSKVGSLENIGHVPGGGQRR
9. IESHKLSFREQAKARTDHGAEIVYQSPNISTDGSPRRLSNVSSSGSINMTDSPQLSTLADQVSASLAKQGL

JAWLESS FISH

**Hagfish** Inshore hagfish

331aa

ENSEBUT00000021644.1, MAP taub

MMGTMVMGRLASFQTLQVTQMAVNKKLNSTMAM

ERRSGLPKPTAIPKPFGIPTSIISQPTTGSMKCI

GYEAQDGMQTQPRGGTPTPPTPGRPGSITGPTTPHERARSGSTTPRRIQAGQK

VAIIRTPSRSPSYHHSQELQ

VAPPMPDVKHVRAKIRSTENLCHHPGGGKIQIFSQKATYG

HVQAKCCSLDNVGHTPRNSQ

VQIQSKKMDFSHVTAKCGSKDNLSFKS

GGGSFQVVSHKINFKDKAKAKVGSLENTSYTPGGGNVK

IESQKLNFREKAQARTDHGADIIICLPAHSSTTTPRRLSNASASGDGPHLSTLAADVSAALAQQGF

**Lamprey** Sea lamprey

277aa

ENSPMAT00000002181.1, MAP

UniPort: S4RAD9

KKGGGAAPVTTPGAAGTVGRPPGSARTSMVPRAPGAPGASRSSATPASGGRGAGATPGGA

SRSVPTTPGEKKASRTLMTPKSPVGTAPNPNLKAVRSKIGSTDNIKHQPAGGK

IQIVTKKLDFSHVTSKCGSMANVKHIPGGGN

VQIVSKKVDVRHVASKCGSKDNITHKPGGGNIQIVS

KKLDFSEKAQSKVGSLDNADHVPGGGNVK

IESQKLSFRETAKSRTNYGNSSHSPTTSEGPSGRMSKVSSTGSLSQDASPEQCDAQEQQNHQQSQQQQ

**INVERTEBRATES**

**Drosophila melanogaster** tau, isoform A

361aa

NCBI: NP_651575.1

1. MADVLEKSSLLDAVPPLGDPHPPLPHQQLQQEAAAAAAANAAPPAPPQQQQPPPHQLQQQQPQQQQL
2. QQKPANARANQDQK
3. EGDNDSGVDESTQEK
4. DRNGPNSPSSPVKTPTSTSSKPDKSGTSRPPSATPSNKSAPKSRSASKNRLLLKTPEPEPVKK
5. VPMNKVQVGHAPSPNLKAVRSKIGSLDNATYKPGGGHVKIESKKIDIKAAPRIEAKNDKYMPKGGEKK
6. IVTTKLQWNAKSKIGSLENAAHKPGGGDKKIETLKMDFKDKAKPKVGSTANVKHQPGGGDIK
7. IQTQKLEIKAQSKVGSLDNVKHKPGGGEKKIFDDKDYLKNVEHSVALTTPPTQ
8. SPLPSMTASGADENLNQQS

A very similar transcript Ensembl FBtr0344743 tau-RC

UniProt: A0A0B4KHQ8

249aa

1. MADVLEKSSLLDAVPPLGDPHPPLPHQQLQQEAAAAAAANAAPPAPPQQQQPPPHQLQQQ
2. QPQQQQLQQKPANARANQDQK
3. EGDNDSGVDESTQEK
4. DRNGPNSPSSPVKTPTSTSSKPDKSGTSRPPSATPSNKSAPKSRSASKNRLLLKTPEPEPVKK
5. VPMNKVQVGHAPSPNLKAVRSKIGSLDNATYKPGGGHVKIESKKIDIKAAPRIEAKNDKYMPKGGEKK
6. IVTTKLQWNAKSKIGSLENAAHKPGGGDKKIETLKMDFKDKAKPKVGSTANVKHQPGGGDIK
7. IQTQKLEIKAQSKVGSLDNVKHKPGGGEKKIFDDKDYLKNVEHSVALTTPPTQ
8. EYIYNFY

Supplementary Fig. 2: Graphical overview and a phylogenic tree exon 4a generated by the COBALT alignment


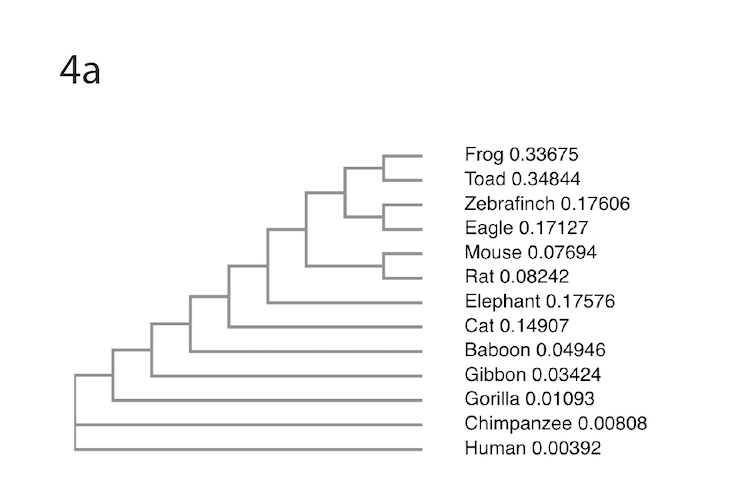


Supplementary Fig. 3: Graphical overview and a phylogenic tree exon 4a-L generated by COBALT alignment


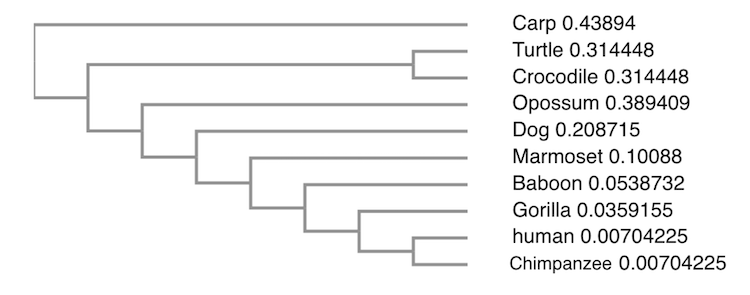


|  |  |  |  |  |  |  |
| --- | --- | --- | --- | --- | --- | --- |

**Supplementary Table 1: N-terminal (exons 1-4) and C-terminal (exons 9-13) homology matrix for primates**

| **Panel a: Exons 1-4** | | | | | | | | |
| --- | --- | --- | --- | --- | --- | --- | --- | --- |
| **Human 776** | 100 | 100 | 100 | 98 | 97 | 95 | 89.74 | 84.68 |
| **Human 833** | 100 | 100 | 100 | 99 | 97 | 94 | 87 | 86 |
| **Chimp** | 100 | 100 | 100 | 98 | 97 | 95 | 89 | 84 |
| **Gorilla** | 98 | 99 | 98 | 100 | 97 | 96 | 89 | 86 |
| **Gibbon** | 97 | 97 | 97 | 97 | 100 | 98 | 89 | 86 |
| **Baboon** | 95 | 95 | 95 | 96 | 98 | 100 | 87 | 88 |
| **Marmoset** | 90 | 87 | 89 | 89 | 89 | 87 | 100 | 83 |
| **Lemur** | 85 | 86 | 85 | 86 | 86 | 88 | 83 | 100 |
|  |  |  |  |  |  |  |  |  |
|  |  |  |  |  |  |  |  |  |
|  |  |  |  |  |  |  |  |  |
| **Panel b: Exons 9-13** | | | | | | | | |
| **Human 776** | 100 | 100 | 100 | 100 | 100 | 99 | 99 | 98 |
| **Human 833** | 100 | 100 | 100 | 100 | 100 | 99 | 99 | 98 |
| **Chimp** | 100 | 100 | 100 | 100 | 100 | 100 | 99 | 98 |
| **Gorilla** | 100 | 100 | 100 | 100 | 100 | 100 | 99 | 98 |
| **Gibbon** | 100 | 100 | 100 | 100 | 100 | 99 | 99 | 98 |
| **Baboon** | 99 | 99 | 100 | 100 | 99 | 100 | 98 | 97 |
| **Marmoset** | 99 | 99 | 99 | 99 | 99 | 98 | 100 | 98 |
| **Lemur** | 98 | 98 | 98 | 98 | 98 | 97 | 98 | 100 |

**Supplementary Table 1:** Analysis of N-terminal and c-terminal of MAPT in primates

Panel a: matrix identity for exons 1-4 (N-terminal). Panel b: matrix identity for exons 9-13 (C-terminal, MTBD).

**Supplementary Table 2: N-terminal (exons 1-4) and C-terminal (exons 9-13) homology matrix for mammals**

|  |  |  |  |  |  |  |  |  |
| --- | --- | --- | --- | --- | --- | --- | --- | --- |
|  |  |  |  |  |  |  |  |  |
| **Panel a: Exons 1-4** | | | | | | | | |
| **Human 776** | 100 | 100 | 86 | 83 | 87 | 83 | 83 | 65 |
| **Human 833** | 100 | 100 | 84 | 81 | 89 | 83 | 81 | 64 |
| **Cat** | 86 | 84 | 100 | 87 | 92 | 81 | 83 | 68 |
| **Elephant** | 83 | 81 | 87 | 100 | 87 | 81 | 82 | 68 |
| **Dog** | 87 | 89 | 91 | 87 | 100 | 82 | 85 | 71 |
| **Rat** | 83 | 83 | 81 | 81 | 82 | 100 | 92 | 66 |
| **Mouse** | 83 | 81 | 83 | 82 | 85 | 92 | 100 | 67 |
| **Opossum** | 65 | 64 | 68 | 68 | 80 | 66 | 67 | 100 |
|  |  |  |  |  |  |  |  |  |
| **Panel b: Exons 9-13** | | | | | | | | |
| **Human 776** | 100 | 100 | 98 | 99 | 98.62 | 97 | 97 | 94 |
| **Human 833** | 100 | 100 | 98 | 99 | 98 | 98 | 98 | 95 |
| **Cat** | 98 | 98 | 100 | 98 | 98 | 97 | 97 | 94 |
| **Elephant** | 99 | 99 | 98 | 100 | 98 | 97 | 97 | 94 |
| **Dog** | 99 | 98 | 98 | 98 | 100 | 98 | 98 | 95 |
| **Rat** | 97 | 98 | 97 | 98 | 98 | 100 | 100 | 95 |
| **Mouse** | 97 | 98 | 97 | 98 | 98 | 100 | 100 | 95 |
| **Opossum** | 94 | 95 | 94 | 95 | 95 | 95 | 95 | 100 |

**Supplementary Table 2:** Analysis of N-terminal and c-terminal of MAPT in mammal

Panel a: matrix identity for exons 1-4 (N-terminal). Panel c: matrix identity for exons 9-13 (C-terminal, MTBD).

**Supplementary Table 3: N-terminal (exons 1-4) and C-terminal (exons 9-13) homology matrix for vertebrates**

|  |  |  |  |  |  |  |  |  |  |  |
| --- | --- | --- | --- | --- | --- | --- | --- | --- | --- | --- |
|  |  |  |  |  |  |  |  |  |  |  |
| **Panel a: Exons 1-4** | | | | | | | | | | |
| **Human 776** | 100 | 100 | 69 | 62 | 61.54 | 61.54 | 37.89 | 32 | 20 | 15.73 |
| **Human 833** | 100 | 100 | 73 | 64 | 61 | 61 | 36 | 28 | 21 | 16 |
| **Zebrafish** | 60 | 73 | 100 | 96 | 85 | 87 | 40 | 34 | 20 | 20 |
| **Eagle** | 62 | 64 | 96 | 100 | 80 | 79 | 34 | 32 | 22 | 25 |
| **Turtle** | 62 | 61 | 85 | 80 | 100 | 84 | 37 | 34 | 21 | 23 |
| **Crocodile** | 62 | 61 | 87 | 79 | 84 | 100 | 38 | 32 | 20 | 25 |
| **Frog** | 38 | 36 | 40 | 34 | 37 | 38 | 100 | 63 | 12 | 15 |
| **Toad** | 32 | 28 | 38 | 32 | 34 | 32 | 63 | 100 | 14 | 11 |
| **Salmon** | 20 | 21 | 20 | 22 | 21 | 20 | 12 | 14 | 100 | 39 |
| **Carp** | 16 | 16 | 20 | 25 | 23 | 25 | 15 | 11 | 39 | 100 |
|  |  |  |  |  |  |  |  |  |  |  |
| **Panel b: Exons 9-13** | | | | | | | | | | |
| **Human 776** | 100 | 100 | 91 | 93 | 92 | 92 | 79 | 81 | 55 | 53 |
| **Human 833** | 100 | 100 | 91 | 93 | 92 | 92 | 79 | 81 | 55 | 53 |
| **Zebrafish** | 91 | 91 | 100 | 98 | 92 | 94. | 80 | 81 | 57 | 55 |
| **Eagle** | 93 | 93 | 98 | 100 | 94 | 95 | 80 | 81 | 57 | 56 |
| **Turtle** | 92 | 92 | 92 | 94 | 100 | 92 | 80 | 83 | 55 | 50 |
| **Crocodile** | 92 | 92 | 94 | 95 | 92 | 100 | 79 | 80 | 55 | 52 |
| **Frog** | 80 | 79 | 80 | 80 | 80 | 79 | 100 | 87 | 53 | 53 |
| **Toad** | 81 | 81 | 81 | 81 | 83 | 80 | 87 | 100 | 55 | 54 |
| **Salmon** | 55 | 55 | 57 | 57 | 55 | 55 | 53 | 55 | 100 | 68 |
| **Carp** | 53 | 53 | 55 | 56 | 50 | 52 | 53 | 54 | 68 | 100 |

**Supplementary Table 3:** Analysis of N-terminal and c-terminal of MAPT in primates

Panel a: matrix identity for exons 1-4 (N-terminal). Panel c: matrix identity for exons 9-13 (C-terminal, MTBD)
